# Metabolic synthetic lethality by targeting NOP56 and mTOR in *KRAS*-mutant lung cancer

**DOI:** 10.1186/s13046-022-02240-5

**Published:** 2022-01-17

**Authors:** Zhang Yang, Shun-Qing Liang, Liang Zhao, Haitang Yang, Thomas M. Marti, Balazs Hegedüs, Yanyun Gao, Bin Zheng, Chun Chen, Wenxiang Wang, Patrick Dorn, Gregor J. Kocher, Ralph A. Schmid, Ren-Wang Peng

**Affiliations:** 1 Division of General Thoracic Surgery and Department of BioMedical Research (DBMR), Inselspital, Bern University Hospital, University of Bern, Murtenstrasse 28, 3008 Bern, Switzerland; 2 Current address: University of Massachusetts Medical School, Worcester, MA 01605 USA; 3 Current address: Department of Thoracic Surgery, Shanghai Chest Hospital, Shanghai Jiao Tong University, Shanghai, 200030 China; 4 Department of Thoracic Surgery, University Medicine Essen – Ruhrlandklinik, University Duisburg-Essen, Essen, Germany; 5 Department of Thoracic surgery, Fujian Medical University Union Hospital, Fuzhou City, Fujian China; 6 Thoracic Surgery Department 2, Hunan Cancer Hospital and The Affiliated Cancer Hospital of Xiangya School of Medicine, Central South University, Changsha, Hunan China

**Keywords:** *KRAS*-mutant cancer, NOP56, mTOR, ROS, Synthetic lethal vulnerability

## Abstract

**Background:**

Oncogenic *KRAS* mutations are prevalent in human cancers, but effective treatment of *KRAS*-mutant malignancies remains a major challenge in the clinic. Increasing evidence suggests that aberrant metabolism plays a central role in *KRAS*-driven oncogenic transformation. The aim of this study is to identify selective metabolic dependency induced by mutant *KRAS* and to exploit it for the treatment of the disease.

**Method:**

We performed an integrated analysis of RNAi- and CRISPR-based functional genomic datasets (*n* = 5) to identify novel genes selectively required for *KRAS*-mutant cancer. We further screened a customized library of chemical inhibitors for candidates that are synthetic lethal with NOP56 depletion. Functional studies were carried out by genetic knockdown using siRNAs and shRNAs, knockout using CRISPR/Cas9, and/or pharmacological inhibition, followed by cell viability and apoptotic assays. Protein expression was determined by Western blot. Metabolic ROS was measured by flow cytometry-based quantification.

**Results:**

We demonstrated that nucleolar protein 5A (NOP56), a core component of small nucleolar ribonucleoprotein complexes (snoRNPs) with an essential role in ribosome biogenesis, confers a metabolic dependency by regulating ROS homeostasis in *KRAS*-mutant lung cancer cells and that NOP56 depletion causes synthetic lethal susceptibility to inhibition of mTOR. Mechanistically, cancer cells with reduced NOP56 are subjected to higher levels of ROS and rely on mTOR signaling to balance oxidative stress and survive. We also discovered that IRE1α-mediated unfolded protein response (UPR) regulates this process by activating mTOR through p38 MAPK. Consequently, co-targeting of NOP56 and mTOR profoundly enhances *KRAS*-mutant tumor cell death in vitro and in vivo.

**Conclusions:**

Our findings reveal a previously unrecognized mechanism in which NOP56 and mTOR cooperate to play a homeostatic role in the response to oxidative stress and suggest a new rationale for the treatment of *KRAS*-mutant cancers.

**Supplementary Information:**

The online version contains supplementary material available at 10.1186/s13046-022-02240-5.

## Background

Oncogenic mutations in the *RAS* family (*HRAS*, *KRAS*, and *NRAS*) are the most common genetic alterations across human cancers and occur in approximately 25% of all tumors (COSMIC; http://cancer.sanger.ac.uk/cosmic). KRAS is the predominant isoform of the RAS family proteins activated by mutations (most frequently at codon 12, 13, and 61) in cancers and is responsible for 85% of all RAS-driven cancers, particularly pancreatic, colon, and non-small cell lung cancer (NSCLC) [1]. Mutant KRAS is associated with poor prognosis and treatment resistance. However, unlike NSCLC with less frequent oncogenic drivers (e.g., EGFR, ALK, MET1, and ROS1) that respond significantly to selective kinase inhibitors [2], effective therapies specifically targeting *KRAS*-mutant cancers remains a challenge [2, 3]. Despite recent progress of immune checkpoint inhibitors of programmed death 1 (PD1) and the ligand PD-L1 in treating NSCLC, they fail to discriminate *KRAS*-mutant from other NSCLC [4]. Covalent KRAS inhibitors have demonstrated promise in preclinical models, but they are only effective for a specific *KRAS-G12C* mutant allele and additional agents are needed to optimize the anticancer efficacy [5–7]. Targeting KRAS downstream effectors, such as the mitogen-activated protein kinase (MAPK) RAF/MEK/ERK, has been widely pursued, but the pleiotropic nature and complex interplay among individual signaling cascades and toxicity ensuing from sustained inhibition of multiple KRAS effector pathways has hindered the translational potential of the strategy [8, 9]. Consequently, identification of new targets for innovative treatment strategies tailored to *KRAS*-mutant cancers still represents a pressing need [3].

The concept to target KRAS synthetic lethality, premised by the notion that oncogenic KRAS signaling fuels a unique cell state, manifested by adaptation to oncogenic stress and transcriptional, translational and metabolic reprogramming, and that interfering with this *KRAS*-driven cell state may result in selective cytotoxicity for *KRAS*-mutant cancer, provides an alternative strategy for treating *KRAS*-driven cancers [10, 11]. Indeed, exploiting cancer cell vulnerabilities contextually induced by mutant KRAS, in particularly the mechanisms critical for surveillance of oncogene-dependent cellular stresses (genotoxic, proteotoxic, and metabolic) that are permissive for strong oncogenic signaling, has not only provided promising therapeutic avenues but also a wealth of information on the fundamental principles of KRAS-induced tumorigenicity [12–14]. Activating *KRAS* mutations deregulate mitosis, nuclear export, redox, and mitochondrial activity, and *KRAS*-mutant cancer cells have consequently been shown to have a greater dependency on the functions of non-oncogenes [e.g., PLK1, XPO1, and MRPL52 (a component of the mitochondrial large ribosomal subunit)] that play critical roles in their respective processes [12–16], suggesting that targeting non-oncogene addiction is an attractive approach for the treatment of *KRAS*-mutant cancer [17, 18].

NOP56 (nucleolar protein 5A or NOL5A) is a ribonuclear protein, which, together with fibrillarin (FBL), NOP58 (nucleolar protein 58), and nonhistone chromosome protein 2-like 1 (NHP2L1 or SNU13p, 15.5 kDa), forms the core protein set of box C/D small nucleolar ribonucleoprotein complexes (snoRNPs) that play an essential role in ribosome assembly by methylating rRNA at the 2′-O-ribose and modulating ribosomal RNA (rRNA) processing [19, 20]. Recent evidence suggests that NOP56 and the other snoRNPs are the novel group of nucleolar proteins that promote cell transformation and tumorigenesis [21, 22]. Indeed, ribosome biogenesis is the only cellular process in which a large number of genes harbor evolutionarily conserved MYC-binding sites [21]. NOP56 is overexpressed in Burkitt’s lymphoma and other cancers and serves as a marker of poor prognosis [23]. In particular, NOP56 is required for MYC-induced cell transformation and tumor growth in Burkitt’s lymphoma [21]. NOP56 may also have extra-ribosomal functions that remain to be discovered. Nevertheless, the activity of snoRNPs in oncogenic transformation suggests that they are promising therapeutic targets for cancer treatment [24, 25].

In this study, we reported an unexpected function of NOP56 in metabolic stress response and a previously unrecognized metabolic synthetic lethality by targeting NOP56 and mTOR in *KRAS*-mutant cancers. Based on integrated analyses of RNAi- and CRISPR-mediated functional genomics [12, 16, 26], we identified NOP56 as a novel metabolic dependency of *KRAS*-mutant cancer by regulating homeostasis of reactive oxygen species (ROS) that plays a well-established role in mutant KRAS-induced tumorigenesis [27–30]. Depletion of *NOP56* impairs the response to oxidative stress, which renders *KRAS*-mutant cancer cells highly dependent on mTOR signaling for survival and particularly vulnerable to mTOR inhibition. Consequently, co-targeting NOP56 and mTOR enhances apoptotic death of *KRAS*-mutant lung cancer cells in vitro and in vivo. We further delineated that mTOR activation upon NOP56 depletion is mediated by IRE1α-mediated unfolded protein response (UPR). These results uncover a previously unknown mechanism by which NOP56 cooperates with UPR and mTOR to regulate metabolic stress and a novel synthetic lethal strategy for the treatment of *KRAS*-mutant cancers.

## Materials and methods

### Cell culture and reagents

Cancer cell lines used in this study (Table S1) were obtained from American Type Culture Collection (ATCC, Manassas, VA, USA). Cells were cultured in RPMI-1640 medium or Medium 199 (Cat. #8758 and #4540; Sigma-Aldrich, St. Louis, MO, USA) supplemented with 10% fetal bovine serum/FBS (Cat. #10270–106; Life Technologies, Grand Island, NY, USA) and 1% penicillin/streptomycin solution (Cat. #P0781, Sigma-Aldrich). The cells were authenticated by DNA fingerprinting and confirmed free from mycoplasma contamination (Microsynth, Bern, Switzerland). All inhibitors used in this study were listed in Table S2.

PF139 and PF563 lung cancer cells were established from lung adenocarcinoma malignant pleural effusion and pleural carcinosis specimens of a 67 year-old female patient and a 75 year-old male patient, respectively, at the time of diagnosis prior to any treatment [31]. Authentication was performed by SNP based cell identification (Multiplexion, Heidelberg, Germany).

### Cell viability and clonogenic survival assay

Lung cancer cells seeded in 96-well plates (2500 cells/well) were dosed 24 h later with different inhibitors for 72 h. Cell viability was determined by PrestoBlue (PB) Cell Viability Reagent (ThermoFisher Scientific) by following the manufacturer’s instructions [14, 31]. The PB reagent was added into media directly (1:10 dilution) and incubated for 30 min-2 h and then the fluorescence was read (excitation 570 nm; emission 600 nm) at recommended time of incubation. The efficacy of drugs on cell growth was normalized to untreated control. Each data point was generated in triplicate and each experiment was done three times (*n* = 3). Best-fit curve was generated in GraphPad Prism [(log (inhibitor) vs response (−variable slope four parameters)]. Error bars are mean ± SD. The combination index (CI) was calculated by ComboSyn software (ComboSyn Inc., http://www.combosyn.com/).

Clonogenic assay was done as we described previously [14, 31–33]. In brief, cells seeded in 6-well plates (3000 cells/well) were dosed 24 h later and continually treated with rapamycin for 7 days (refresh drugs every 3 days), the resulting colonies were stained with crystal violet (0.5% dissolved in 25% methanol).

### Apoptosis assays

Lung cancer cells were treated for 72 h with vehicle or rapamycin. After treatment, cells in the supernatant and adherent to plates were collected, washed with PBS and pooled before suspended in binding buffer and stained with the Annexin V Apoptosis Detection Kit -FITC (Cat. #88–8005; Thermo Fisher Scientific, Waltham, MA, USA) according to the manufacturer’s instructions. Flow cytometry analysis was performed on a BD Biosciences LSRII flow cytometer.

### Gene silencing by small interfering (siRNA), short hairpin RNAs (shRNA) and single-guide RNAs (sgRNA)

Transient knockdowns were mediated by siRNAs. Cells cultured in triplicate at 50–70% confluency were transfected using SiTran1.0 (TT300001; Origene Technologies, Rockville, MD, USA) according to the manufacturer’s protocol. *NOP56* (CAT#: SR307156), *EIF4E* (CAT#: SR320018), *RPS6* (CAT#: SR304160)*, RAPTOR* (CAT#: SR324724)*,* and *RICTOR* (CAT#: SR326062) were knocked down by specific pooled siRNA duplexes purchased from OriGene Technologies, with control siRNA Duplex as a negative control.

Stable knockdown of *NOP56* was achieved via lentiviral delivery of *NOP56* Human shRNA Plasmid Kit (SHCLND_006392, MERCK). A scramble shRNA was used as a control. Lentiviral particles were generated and cells infected according to the protocol from Broad Institute. The supernatant containing lentiviruses was collected, filtered through 0.45 μM filters, and stored in aliquots at − 80 °C, or immediately used to infect recipient cells. After infection, cells were selected in puromycin (1.5 μg/ml) and further passaged in culture for functional assays. *NOP56* knockout was performed via a CRISPR/Cas9 and non-homology mediated approach using the NOL5A (NOP56) Human Gene Knockout Kit (CAT#: KN411153; OriGene Technologies) according to the manufacturer’s protocol.

### Quantitative real-time PCR (qRT-PCR)

Total RNA was isolated and purified using RNeasy Mini Kit (Qiagen, Hilden, Germany). Complementary DNA was synthesized by the High capacity cDNA reverse transcription kit (Applied Biosystems, Foster City, CA, USA) according to manufacturer’s instructions. Real time PCR was performed in triplicate on a 7500 Fast RealTime PCR System (Applied Biosystems) using TaqMan primer/probes (Applied Biosystems): *HSPA5*, Hs00607129_gH; *ERN1*, Hs00980095_m1; *EIF2AK3*, Hs00984003_m1; *ATF4*, Hs00909569_g1; *DDIT3*, Hs00358796_g1, with *GAPDH* (Hs02786624_g1) and *ACTB* (Hs01060665_g1) used as endogenous normalization controls.

### Immunoblotting, immunohistochemistry and immunofluorescence

Cell lysates were prepared and western blot analysis was performed as described [14, 31]. In brief, equal amounts of protein lysates resolved by SDS-PAGE (Cat. #4561033; Bio-Rad Laboratories, Hercules, CA, USA) and transferred onto nitrocellulose membranes (Cat. #170–4158; Bio-Rad). Membranes were then blocked with blocking buffer (Cat. #927–4000; Li-COR Biosciences, Bad Homburg, Germany) for 1 h at room temperature (RT) and incubated with appropriate primary antibodies overnight at 4 °C (Table S3). IRDye 680LT-conjugated goat anti-mouse IgG (Cat. #926–68,020) and IRDye 800CW-conjugated goat anti-rabbit IgG (Cat. #926–32,211) from Li-COR Biosciences were used at 1:5000 dilutions. Finally, signals of membrane-bound secondary antibodies were imaged using the Odyssey Infrared Imaging System (Li-COR Biosciences).

For immunofluorescence, tumor cells grown on poly-lysine-treated coverslides were fixed with 4% paraformaldehyde for 15 min at RT and permeabilized with cold methanol (− 20 °C) for 5 min or with 0.1% Triton X-100/PBS at RT for 15 min before incubated overnight at 4 °C with primary antibodies (Table S3). The cells were incubated for 1 h at RT with Alexa Fluor 647 goat anti-mouse IgG (Cat. #A21236) or Alexa Fluor 488 goat anti-Rabbit IgG (Cat. #A11034) from Invitrogen (Eugene, OR, USA). Nuclei were counterstained by 4′,6-diamidino-2-phenylindole. Images were acquired on a ZEISS Axioplan 2 imaging microscope (Carl Zeiss MicroImaging, Göttingen, Germany) and processed using Adobe Photoshop CS6 v.13 (Adobe Systems, San Jose, CA, USA).

Immunohistochemical study was performed as we described previously [31, 32]. In brief, surgically removed xenograft tumors were formalin-fixed and paraffin-embedded (FFPE). FFPE tumors were sectioned at 4 μm, deparaffinized, rehydrated and subsequently stained with hematoxylin and eosin (H&E) and appropriate antibodies (Table S3) using the automated system BOND RX (Leica Biosystems, Newcastle, UK). Visualization was performed using the Bond Polymer Refine Detection kit (Leica Biosystems) as instructed by the manufacturer. Images were acquired using PANNORAMIC® whole slide scanners, processed using Case Viewer (3DHISTECH Ltd.). The staining intensities of the whole slide (two tumors/group) were quantified by QuPath software.

### In vivo mouse study

Mouse studies were conducted in accordance with Institutional Animal Care and Ethical Committee-approved animal guidelines and protocols. All mouse experiments were performed in age- and gender-matched NSG (NOD-*scid IL2Rγ*^*null*^) as we previously described [31, 32]. Tumor cells in DMEM (H460-shScrambled or H460-shNOP56) 1:1 mixed with BD Matrigel Basement Membrane Matrix (Cat. #356231; Corning, NY, USA) were subcutaneously inoculated in left and right flanks (0.5 × 10^6^/injection). When tumors were palpable, mice were randomly assigned to treatment groups: 1) control; 2) rapamycin (0.1 mg/kg, i.p, 5 days/week) for 5 weeks. Tumors were measured every 3 days, with their size calculated as follows: (length × width^2^)/2. For survival analysis, the mice were closely monitored on a daily basis, and the size of tumors was measured with a caliper every 4–5 days. Mice were sacrificed when the tumor volume reached 1500 mm^3^.

### Public databases

**Fig. 1:**
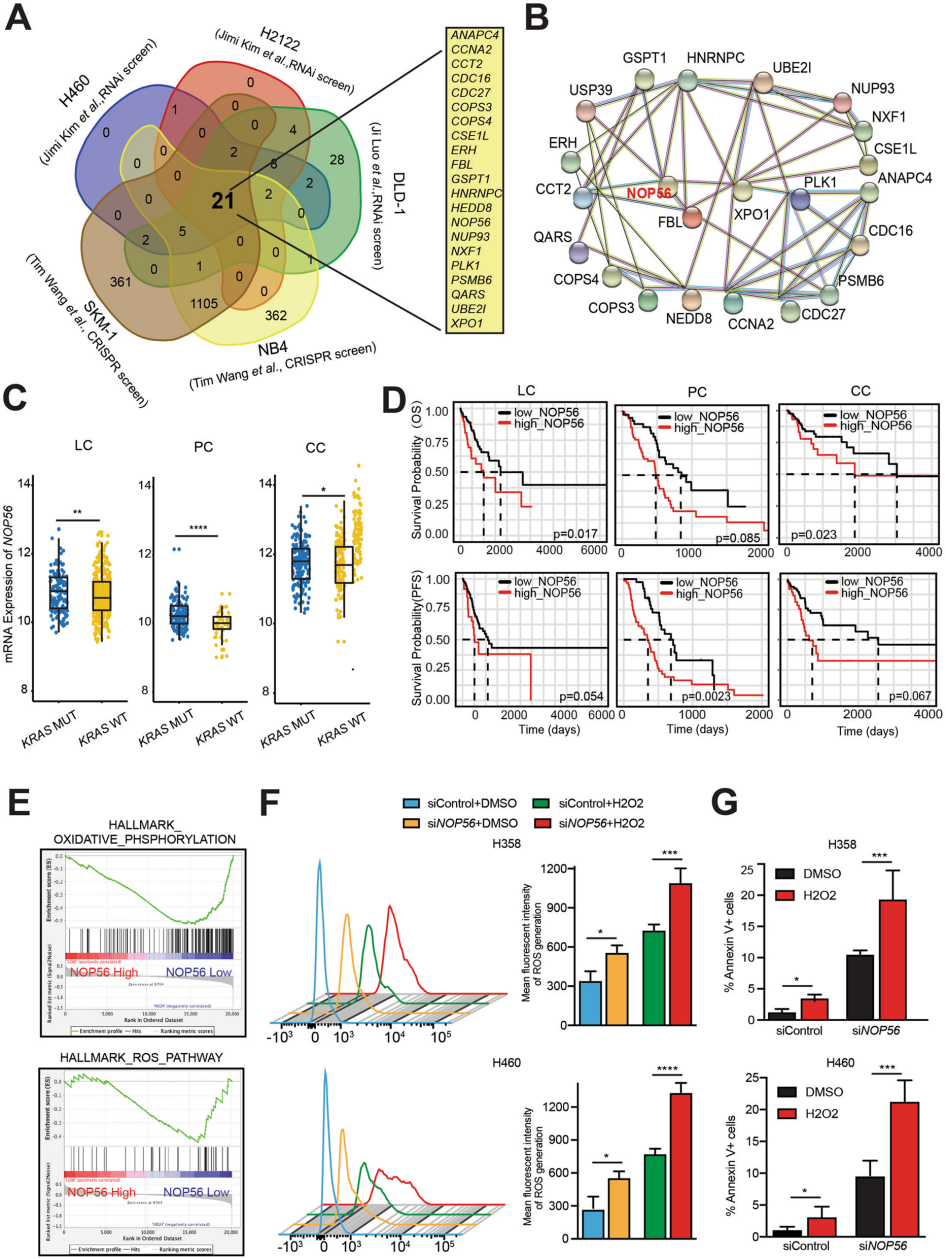
NOP56 confers a metabolic dependency in *KRAS*-mutant cancers. **A**, Venn diagram showing common essential genes in *KRAS*-mutant cancer cells. Data are based on the published studies, with the 21 common genes listed on the right. **B**, Network analysis of the 21 common genes by STRING. **C**, *NOP56* mRNA expression in *KRAS*-mutant lung cancer (LC), pancreatic cancer (PC) and colon cancer (CC) versus *KRAS*-wild-type (WT) cancers in patient samples from TCGA. **D**, Prognostic values of *NOP56* expression across TCGA lung adenocarcinoma (left), pancreatic cancer (middle) and colon cancer (right) cohorts harboring *KRAS* mutations. Kaplan–Meier survival analyses were stratified by the optimal cut-off value of *NOP56* mRNA levels. **E**, Gene set enrichment analysis (GSEA) revealed significant enrichment of oxidative phosphorylation and ROS pathway gene signatures in *NOP56*-depleted *KRAS*-mutant cancer cells (SW480). The GEO dataset GSE15212 was used for GSEA. **F,** H358 and H460 cells transfected with *NOP56-*specific or control siRNAs were treated (72 h post-transfection) with 300 μM H_2_O_2_ for 6 h, followed by incubation with H2DCFDA for 30 min, and analyzed by flow cytometry. Quantification of relative ROS levels was shown in the right. Data are shown as the mean ± SD (*n* = 3). *P < 0.05,***P < 0.001, ****P < 0.0001 by two-way ANOVA with Tukey’s multiple comparisons test. **G**, H358 and H460 cells transfected with *NOP56*-specific or control siRNAs were subsequently (72 h post transfection) treated with vehicle (DMSO) or 300 μM H_2_O_2_ for 6 h before apoptotic assay. Data were shown as mean ± SD (*n* = 3). *P < 0.05 and ***P < 0.001 by two-way ANOVA with Tukey’s multiple comparisons test

To identify synthetic lethal targets in *KRAS*-mutant cancers, we interrogated functional genomics dataset of CRISPR/Cas9 knockout and RNAi/shRNA knockdown screens from published studies: whole genome RNAi screens in DLD-1 colon cancer cells [12] and in *KRAS*-mutant lung cancer cells (H2122, H2009, HCC44, H460, H1155) [16], genome-wide CRISPR/Cas9 loss of function screens in *KRAS*-mutant leukemia cells (PL-21, SKM-1, NB4) [26]. To minimize the effects of cancer lineage and histological subtype, we selected DLD-1 (colon), H460 (large cell lung carcinoma), H2122 (lung adenocarcinoma), SKM-1 (without *PML-RARA* fusion) and NB4 (*PML-RARA*) for further analysis, which identified a number of common candidates (*n* = 21) as *KRAS* synthetic lethal partners (Fig. 1).

Interrogation of publicly available datasets was performed as we have described [14, 31]. Specifically, transcriptomic data of lung, pancreatic and colon cancer were obtained from the Cancer Genome Atlas (TCGA) (https://portal.gdc.cancer.gov/projects/TCGA). Gene set enrichment analysis (GSEA) was performed by using GSEA software. The transcriptomic dataset (GSE15212) used for GSEA was derived from *KRAS*-mutant colon cancer cell line (SW480) treated with *NOP56*-specific siRNAs and downloaded from the Gene Expression Omnibus (GEO) database. For survival analysis, transcriptomic gene expression and corresponding survival data were extracted and analyzed by using the “maxstat”, “survival”, and “survminer” packages in R software (version 3.6.0). Patients were divided into two groups (high_ *NOP56* versus low_ *NOP56*) based on the optimal cutoff value of *NOP56* transcripts across all patients to plot the Kaplan–Meier survival curves.

For correlative analysis of *NOP56* expression with sensitivity (IC_50_) to mTOR inhibitors, gene expression data and drug response profiles were downloaded from Cancer Cell Line Encyclopedia (CCLE) and Genomics of Drug Sensitivity in Cancer (GDSC) databases, respectively. Correlation analysis was performed using R software (version 3.6.0).

### Statistical analysis

Statistical analyses were performed using GraphPad Prism 7.01 (GraphPad Software Inc., San Diego, CA, USA) unless otherwise indicated. In all studies, data represent biological replicates (n) and are depicted as mean values ± SD or mean values ± SEM as indicated in the figure legends. In all analyses, *P* values less than 0.05 were considered statistically significant. For the survival analysis, patients were grouped by gene expression, where ‘high’ and ‘low’ expression groups were stratified by the optimal cut-off value.

## Results

### NOP56 confers a metabolic dependency by regulating ROS homeostasis in *KRAS*-mutant lung cancer

To identify therapeutic vulnerabilities in *KRAS*-mutant cancers, we performed integrated analysis of shRNA- and CRISPR-based functional genomics of previously published studies [12, 16, 26]. To minimize lineage-specific effects, we analyzed whole-genome dropout screen dataset in *KRAS-*mutant lung (H460, H2122), colon (DLD-1), acute promyelocytic leukemia (NB4) and acute myeloid leukemia (SKM-1) cancer cells, which identified 21 common genes whose loss of function is synthetic lethal with mutant *KRAS* alleles in distinct cancer lineages (Fig. 1A; Table S4). The protein products of these genes fall into several functional categories, with FBL, NOP56, PLK1 and XPO1 as a core set based on their interaction network (Fig. 1B). Remarkably, PLK1 and XPO1 have been reported to be selectively required for *KRAS*-mutant cancers by counteracting mitotic and nuclear export stress associated with KRAS-induced tumorigenesis [12, 16], and our recent study has implicated PLK1 in metabolic stress response of *KRAS*-mutant cancers [14]. FBL has also been assigned as a promising target in cancers [34, 35], suggesting the power of functional genomics in identifying oncogene-specific vulnerabilities and the accountability of our analyses. In the present study, we investigated the function of NOP56 in *KRAS*-mutant cancers.

Our investigations began with NOP56 knockdown using small interfering RNAs (siRNAs), which revealed that downregulation of NOP56 significantly inhibited the proliferation of numerous *KRAS-*mutant lung (H358, H460, A549, PF563, PF139), pancreatic (MIAPaCa, HPAF-II) and colon (HCT-116, DLD-1) cancer cells, which differ not only in tumor lineages and histological subtypes but also in *KRAS* mutations, e.g., G12C, G12D, Q61H, etc. (Fig. S1A, B; Table S1). Notably, NOP56 silencing also inhibited *NRAS*-mutant lung cancer H1299 cells, although the effects on *EGFR*-mutant (EBC-1) or *FGFR1*-amplified (H520) lung cancer cells were negligible (Fig. S1A, B). Supporting these observations, *KRAS*-mutant lung, pancreatic and colon cancer showed significantly higher expression of *NOP56* than *KRAS*-WT tumors (Fig. 1C) and patients with *KRAS*-mutant lung adenocarcinoma, pancreatic and colon cancer characterized by a higher *NOP56* level are associated with significantly shorter survival (Fig. 1D). In contrast, *NOP56* expression is not a prognostic marker for *KRAS*-mutant lung, pancreatic and colon cancers (Fig. S1C). These results indicate a unique function for NOP56 in *KRAS*-mutant cancers.

To explore NOP56 functions in *KRAS*-mutant malignancies, we profiled the transcriptomic gene expression data of a previous study [36], whereby *NOP56* in *KRAS*-mutant colon cancer cells (SW480) was silenced by siRNAs. Our analysis revealed that high expression of *NOP56* was positively correlated with the gene signature of KRAS signaling (Fig. S1D), in line with the above results (Fig. 1A-D; Fig. S1A, B), and that, importantly, siRNA-mediated *NOP56* knockdown led to significant enrichment of the gene sets involved in ROS pathway (consisting of 49 genes upregulated by ROS) and oxidative phosphorylation (a set of 200 genes encoding proteins involved in oxidative phosphorylation), the latter representing a major source of ROS production (Fig. 1E), suggesting a possible role for NOP56 in the suppression of metabolic ROS that is critical for *KRAS*-induced tumorigenesis [27–30]. Supporting this notion, NOP56 knockdown (KD) by siRNAs significantly upregulated ROS in H358 and H460 cells, and H_2_O_2_ treatment, which elevated the already high level of oxidative stress, provoked significantly greater apoptosis in *NOP56* KD H358 and H460 cells than the control counterparts (Fig. 1F, G). These results uncover NOP56 as a metabolic dependency in *KRAS*-mutant cancer by exerting a previously unrecognized role in the surveillance of oxidative stress.

### NOP56 suppression evokes IRE1α-mediated UPR to mitigate oxidative stress

**Fig. 2:**
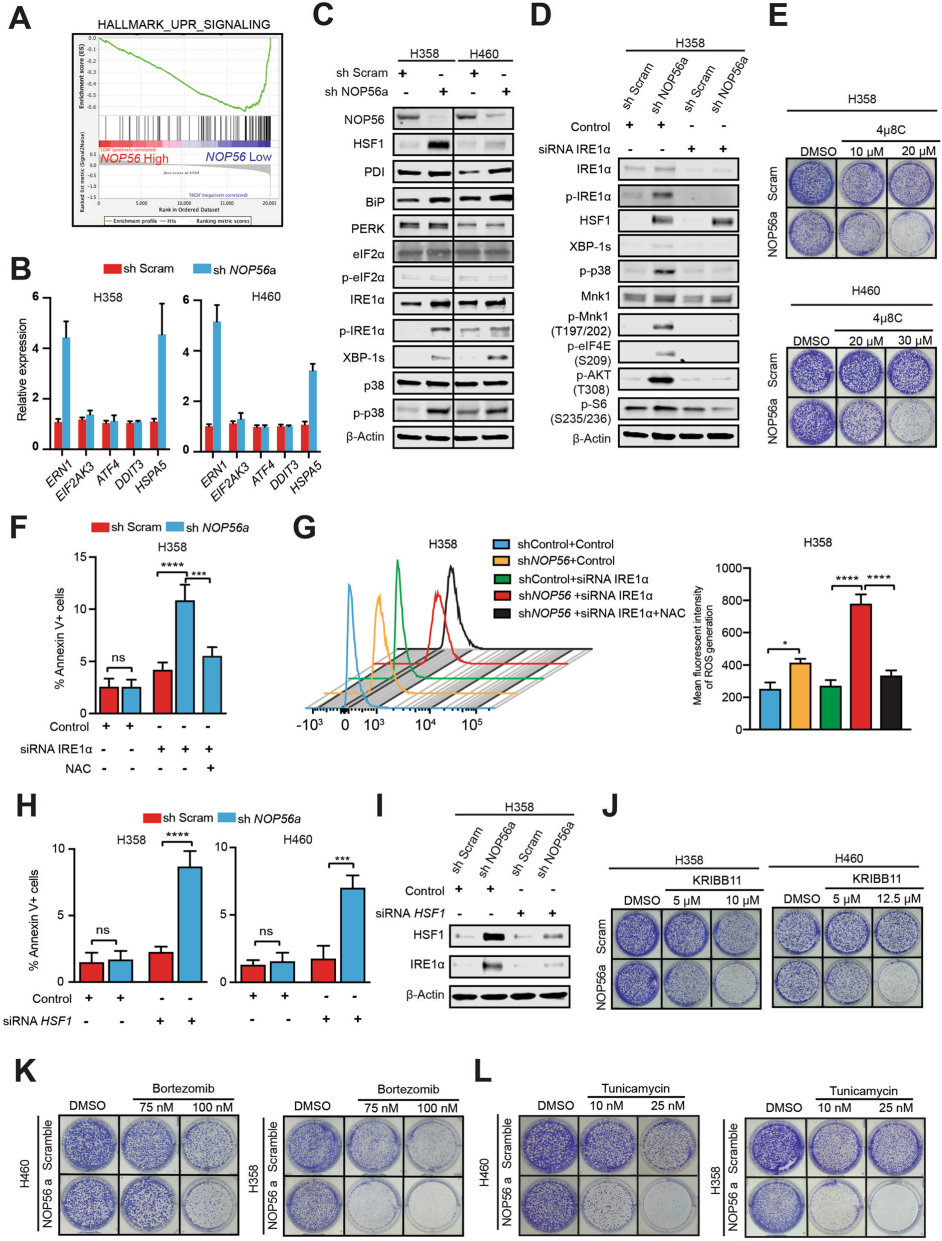
NOP56 depletion evokes IRE1α-mediated UPR. **A**, *NOP56* depletion led to significant enrichment of the UPR gene signature in *KRAS*-mutant cancer cells. GSEA was based on the GEO dataset GSE15212. **B**, Transcriptional quantification (qRT-PCR) of UPR genes in H358 and H460 cells expressing control (sh Scram) or *NOP56*-specific shRNA (sh *NOP56*a). **C**, Immunoblots of H358 and H460 cells expressing scrambled control or *NOP56*-specific shRNAs. **D**, H358 cells expressing scrambled control or *NOP56*-specific shRNAs were transfected with *IRE1α-*specific or control siRNAs for 72 h before immunoblotting. **E**, Clonogenic assay of H460 and H358 cells expressing scrambled control or *NOP56*-specific shRNAs after treated with indicated doses of 4μ8C (IRE1α inhibitor). Representative images are shown. **F**, H358 cells expressing scrambled control or *NOP56*-specific shRNAs were transfected with *IRE1α-*specific or control siRNAs for 72 h, in the presence or absence of NAC (2.5 mM) before apoptosis assay. Data are presented as mean ± SD (*n* = 3). ***P < 0.001, ****P < 0.0001 and ns *P*>0.05 by two-way ANOVA with Tukey’s multiple comparisons test. **G**, H358 cells expressing scrambled control or *NOP56*-specific shRNAs were transfected with *IRE1α-*specific or control siRNAs for 72 h, in the presence or absence of NAC (2.5 mM). Cells were then washed, incubated with H2DCFDA for 30 min, and analyzed by flow cytometry. Quantification of relative ROS levels was shown in the right. Data are presented as mean ± SD (*n* = 3). ***P < 0.001, ****P < 0.0001 and ns *P*>0.05 by two-way ANOVA with Tukey’s multiple comparisons test. **H**, H358 cells expressing scrambled control or *NOP56*-specific shRNAs were transfected with *HSF1-*specific or control siRNAs for 72 h before apoptotic assay. **I**, H358 cells expressing scrambled control or *NOP56-*specific shRNAs were transfected with *HSF1-*specific or control siRNAs for 72 h before immunoblot analysis. Data are presented as mean ± SD (*n* = 3). ***P < 0.001, ****P < 0.0001 and ns *P*>0.05 by two-way ANOVA with Tukey’s multiple comparisons test. **J**, Clonogenic assay of H460 and H358 cells expressing scrambled control or *NOP56*-specific shRNAs after treated with the indicated doses of KRIBB11 (HSF1 inhibitor). Representative images are shown. **K**, **L**, Clonogenic assay of H460 and H358 cells expressing scrambled control or *NOP56*-specific shRNAs after treated with the indicated doses of the ER stress inducer bortezomib (K) or tunicamycin (L). Representative images are shown

Next, we investigated the mechanism that *KRAS*-mutant cancer cells utilize to orchestrate cytotoxic ROS upon NOP56 depletion. GSEA of transcriptomic dataset [36] revealed that *NOP56* knockdown significantly enriched the genes involved in the unfolded protein response/UPR (a set of 113 genes upregulated during UPR) in *KRAS*-mutant cancer cells (Fig. 2A), suggesting that tumor cells might engage the UPR to protect from *NOP56* KD-induced surge of cytotoxic ROS. To test this possibility, we knocked down NOP56 in *KRAS*-mutant lung cancer cells (H358, H460) by using short-hairpin RNAs (shRNA) (Fig. S2A, B). In contrast to the results from siRNA-mediated acute depletion, stable expression of two independent shRNAs showed negligible effects on H358 and H460 proliferation (Fig. S2C, D), which may be due to the activation of compensatory mechanisms. Importantly, several UPR genes, in particular *ERN1* and *HSPA5* encoding the ER stress sensor IRE1α and the chaperon protein BiP, respectively, were markedly upregulated in NOP56-depleted H358 and H460 cells (Fig. 2B). Western blot confirmed the increase of BiP, IRE1α, XBP-1 s (IRE1α effector) and of the master UPR transcription factor HSF-1 (heat shock factor 1) and PDI (protein disulfide isomerase), an important ER chaperone induced during ER stress by carrying out a redox reaction and responsible for the formation of disulfide bonds in proteins (Fig. 2C). Notably, p38 MAPK, a key stress-responsive kinase and an UPR effector [37], was highly phosphorylated (activated) in NOP56-depleted H358 cells (Fig. 2C). Moreover, IRE1α KD blunted p-p38, p-AKT (T308), p-MNK1, p-eIF4E and p-S6 in NOP56-depleted H358 cells, indicating that IRE1α-mediated UPR acts upstream of p38 signaling (Fig. 2D).

Importantly, genetic (siRNA) and pharmacological (4μ8C, an inhibitor of IRE1α) inhibition of IRE1α preferentially impaired *NOP56* KD H358 and H460 cells, manifested by significantly greater proliferative inhibition and apoptotic induction in these cells than in control cells (Fig. 2E, F; Fig. S2E, F). Importantly, the increase of IRE1α KD-induced apoptotic cell death was paralleled by ROS upregulation, and addition of NAC, an ROS scavenger largely dampened IRE1α KD-induced apoptosis (Fig. 2F, G), supporting a role for the UPR in response to oxidative stress. Similarly, genetic and pharmacological inhibition (with KRIBB11) of HSF1 suppressed the proliferation and evoked apoptotic cell death to a markedly greater extent in *NOP56* KD H358 cells than in control cells (Fig. 2H-J).

The outcome of UPR ranges from adaptation to apoptosis [38] and, as such, perturbations of ER homeostasis in cells with an already high level of ER stress, e.g., treatment with bortezomib and tunicamycin that induce persistent ER stress by targeting the 26S proteasome and the ER chaperone BiP, respectively, evoke programmed cell death [38, 39]. Indeed, *NOP56* KD H358 and H460 cells with high basal levels of ROS are highly susceptible to bortezomib and Tunicamycin compared to control cells (Fig. 2K, L).

Thus, targeting NOP56 disrupts ROS homeostasis and induces IRE1α-mediated UPR in *KRAS*-mutant lung cancer cells.

### IRE1α-mediated UPR fuels mTOR signaling via p38 MAPK

**Fig. 3:**
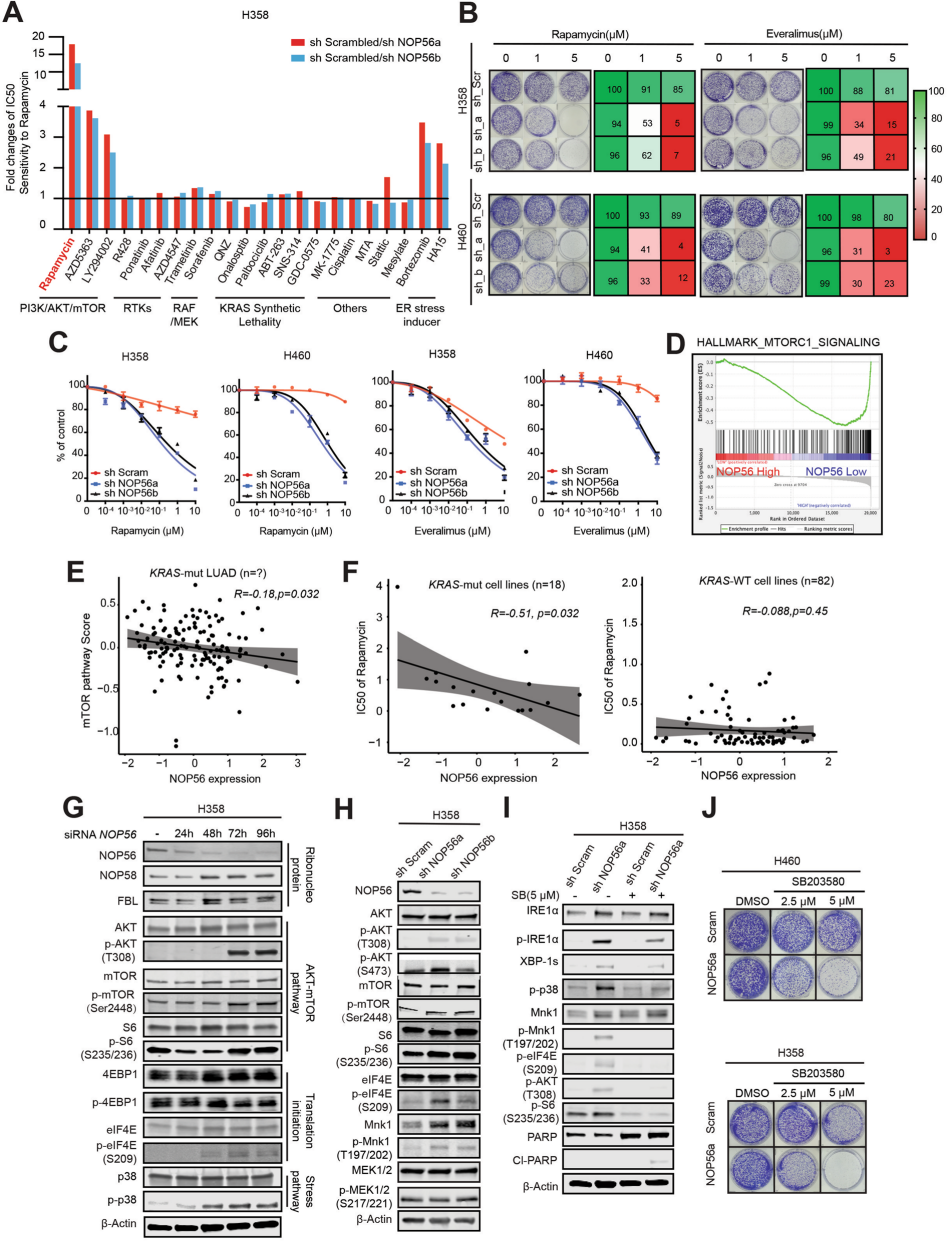
IRE1α-mediated UPR fuels mTOR signaling via p38 MAPK. **A**, H358 cells expressing scrambled control or *NOP56*-specific shRNAs were treated with different inhibitors, with bar graphs illustrating sensitivity increase after *NOP56* KD. Fold changes of IC_50_ values were presented as IC_50_ of rapamycin in H358 cells expressing scrambled shRNA (sh_Scrambled) compared to that in H358 cells expressing *NOP56*-targeted shRNAs (shNOP56 a/b). Data presented as mean (*n* = 2). **B**, Clonogenic assay of H358 and H460 cells expressing control shRNA (sh_Scr) or *NOP56*-specific shRNAs (sh-a, sh-b) after treatment with indicated doses of rapamycin or everolimus. Representative images are shown. The heatmap (right) indicates the percentage of viable cells after the treatment, based on quantification of clonogenic results (left). Data are presented as mean ± SD (*n* = 3). **C**, Growth inhibition of H358 and H460 cells expressing *NOP56*-specific shRNAs (sh NOP56a, sh NOP56b) or control shRNAs (sh Scram) after treated for 72 h with the mTOR inhibitors (rapamycin, everolimus). Data are presented as mean ± SD (*n* = 3). **D**, *NOP56* silencing significantly enriched the mTOR gene signature in *KRAS*-mutant cancer cells. GSEA was performed based on the GEO dataset GSE15212. **E**, Negative correlation of *NOP56* mRNA levels with mTOR gene signature (mTOR pathway score) as determined in a TCGA cohort of patients with *KRAS*-mutant lung adenocarcinoma. Pearson and Spearman coefficient, as well as the significance (*p*-value), were determined using R software (Cor.test function). **F**, *NOP56* expression is a predictive marker of sensitivity (IC_50_) to rapamycin in *KRAS*-mutant cancer cell lines (*n* = 18) but not in *KRAS*-wide-type cancer cell lines (*n* = 82). Drug response profiles were downloaded from the GDSC (Genomics of Drug Sensitivity in Cancer) database. **G,** Immunoblots of H358 cells after transfection with scramble control siRNAs (si-Control) for 72 h (−) or *NOP56*-specific siRNAs (si-*NOP56*) for different time points (24 h, 48 h, 72 h and 96 h). **H**, Immunoblots of H358 and H460 cells expressing scrambled control or *NOP56*-specific shRNAs. **I**, Immunoblots of H358 cells expressing scramble control or the *NOP56*-specific shRNAs after treated with the p38 inhibitor SB203580 (5 μM) for 24 h. **J**, Clonogenic assay of H460 and H358 cells expressing control or *NOP56*-specific shRNAs after treated with the p38 inhibitor SB203580. Representative images are shown

To identify cellular processes that may present therapeutic vulnerabilities in *NOP56* KD cells, we performed synthetic lethal chemical screens with small-molecule drugs (*n* = 22) that interrogate various oncogenic pathways, with the ER stress inducers (bortezomib and HA15) included as positive controls (Table S2). Our screens showed that, except for bortezomib and HA15, LY294002, AZD5363, and rapamycin, inhibitors of the PI3K/AKT/mTOR pathway, preferentially suppressed the viability of *NOP56* KD cells, gauged by their IC_50_ decrease in *NOP56* KD H358 and H460 cells versus control cells (Fig. 3A; Fig. S3A). The greatest change in sensitivity was conferred by rapamycin, which was 0.7 μM and 1.0 μM in H358_shNOP56a and H358_shNOP56b but 12.0 μM in H358_Scr cells, with a selectivity index (IC_50_ in control cells / IC_50_ in *NOP56* KD cells) of 17- and 12-fold, respectively (Fig. 3A; Fig. S3A). These observations were validated by independent assays, in which *NOP56* KD sensitized H358 and H460 cells to PI3K/AKT inhibitors (LY294002, AZD5363), anti-mTOR drugs (rapamycin, everolimus), and ER stress inducers (bortezomib and HA15) (Fig. 3B,C; Fig. S3B,C). Importantly, CRISPR/Cas9-mediated knockout of *NOP56* dramatically increased the sensitivity of *KRAS*-mutant (H358, H460) but not of wild-type (H520, H1703) lung cancer cells to rapamycin (Fig. S3D, E).

Moreover, examining gene expression data of *KRAS*-mutant cancer cells [36] revealed that *NOP56* silencing significantly enriched the mTOR gene signature (Fig. 3D). Mining TCGA and Genomics of Drug Sensitivity in Cancer (GDSC) databases showed that *NOP56* expression is negatively correlated with that of mTOR pathway genes in patients with *KRAS*-mutant lung adenocarcinomas (Fig. 3E) and that *NOP56* mRNA levels are a predictive marker of sensitivity (IC_50_) to rapamycin in *KRAS*-mutant cancer cell lines but not in *KRAS*-wild-type cancer cells (Fig. 3F). These data support our in vitro results (Fig. 3A-C; Fig. S3A-C) and further suggest a reciprocal interplay between NOP56 and mTOR signaling.

Indeed, siRNA-mediated *NOP56* KD, which slightly increased ribonucleolar proteins (e.g., NOP58, FBL), markedly induced AKT/mTOR (p-AKT, p-mTOR, pS6), translation initiation (p-eIF4E) and the stress-responsive p38 MAPK in H358 cells in a time-dependent manner (Fig. 3G), as did shRNA-mediated stable *NOP56* KD in H358 and H460 cells, but not in *KRAS*-WT lung cancer H1703 cells (Fig. 3H; Fig. S3G). Importantly, *NOP56* KD sensitized *KRAS*-mutant lung (A549), colon (HCT-116, DLD-1, and LS174T), pancreatic (MIAPaCa, HPAF-II) and primary *KRAS*-mutant lung cancer cells (PF563, PF139) to rapamycin, as well as *NRAS*-mutant lung cancer H1299 cells but not *KRAS*-wild-type H2405 (BRAF-mutant), EBC-1 (EGFR-mutant), H1993 (MET amplification) and H520 (FGFR1 amplification) cells (Fig. S3H, I). These results reinforce the notion that NOP56 plays a unique role in *KRAS*-mutant cancer.

Our results demonstrated that NOP56 and the IRE1α-mediated UPR act upstream of p38 and mTOR signaling (Fig. 2C, D; Fig. 3G), suggesting a signaling cascade from the UPR to mTOR via p38 MAPK. To confirm this, we targeted p38 with the specific inhibitor SB203580, which, as expected, barely affected the upstream IRE1α-dependent UPR (p-IRE1α, XBP-1 s), but strikingly dampened the MNK-eIF4E axis and mTOR signaling (p-AKT, p-S6) in H358 cells (Fig. 3I). Importantly, SB203580 exposure not only decreased activity of the mTOR pathway but also increased PARP expression and promoted PARP cleavage (Fig. 3I), concurrent with substantially elevated cytotoxicity on *NOP56* KD H460 and H358 cells compared to that on control cells (Fig. 3J). Together, these results unravel a signaling cascade from the IRE1α-mediated UPR to p38 MAPK and to mTOR signaling in *KRAS*-mutant lung cancer upon NOP56 suppression.

### Synthetic lethality by targeting NOP56 and mTOR in *KRAS*-mutant lung cancer

**Fig. 4:**
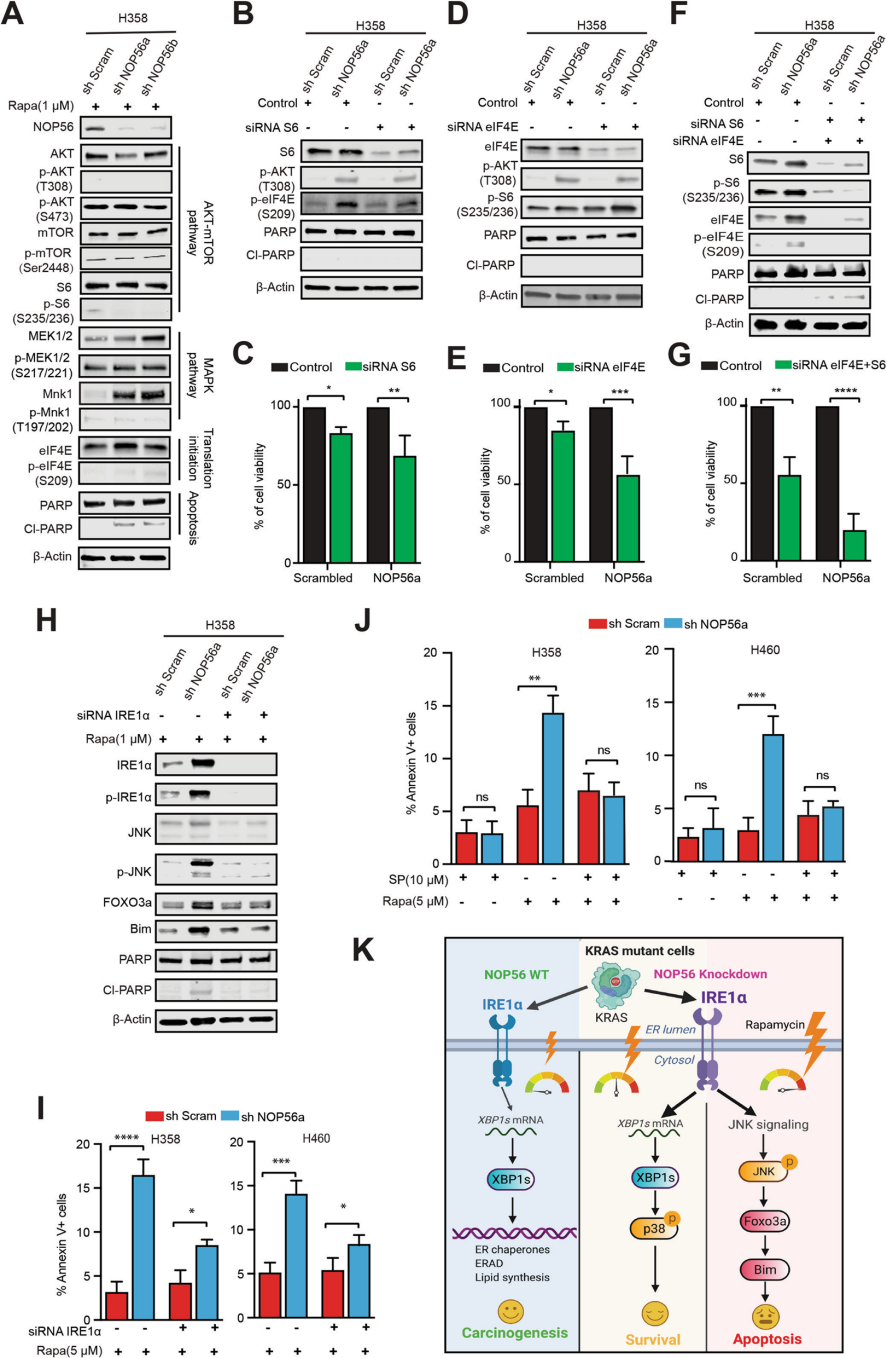
Synthetic lethality by targeting NOP56 and mTOR in *KRAS*-mutant lung cancer cells. **A**, Immunoblots of H358 cells expressing scramble control or *NOP56*-specific shRNAs after treated with rapamycin (1 μM) for 24 h. **B-G** H358 cells expressing scramble control or sh*NOP56*-specific shRNAs were transfected with control siRNAs or the indicated siRNAs specifically targeting S6, eIF4E, alone and in combination. The cells were then subjected to immunoblots (B, D, F) and viability assay (C, E, G) 72 h post-transfection. Data are presented as mean ± SD (*n* = 3). **H**, H358 cells expressing scrambled control or *NOP56*-specific shRNAs were transfected with *IRE1α-*specific or control siRNAs for 48 h, followed by treatment with rapamycin (1 μM) for 24 h before immunoblotting. **I**, H358 cells expressing control or *NOP56-*specific shRNAs were transfected with *IRE1α-*specific or control siRNAs for 24 h, followed by treatment with rapamycin (5 μM) for 72 h before apoptosis assay. Data are presented as mean ± SD (*n* = 3). *p < 0.05, ***P < 0.001 and ****P < 0.0001 by two-way ANOVA with Tukey’s multiple comparisons test. **J**, H358 cells expressing control or *NOP56-*specific shRNAs were preincubated overnight with vehicle (DMSO) or the JNK inhibitor SP600125, followed by treatment with rapamycin for 72 h before apoptosis assay. Data are presented as mean ± SD (*n* = 3). **p < 0.01, ***P < 0.001 and ns *P*>0.05 by two-way ANOVA with Tukey’s multiple comparisons test. **K**, Proposed model of cellular gauge for IRE1α-regulated UPR. In *KRAS*-mutant cancer cells, intact NOP56 keeps ROS in check so that IRE1α-regulated UPR is minimal (basal level; left). Intermediate levels of IRE1α-regulated UPR ensue from NOP56 depletion, which activates p38-AKT/mTOR and promotes cell survival (middle). At “dangerous” level of ROS, IRE1α-regulated UPR initiates JNK-dependent apoptosis (right)

Our findings that *NOP56* KD cells are exposed to higher levels of metabolic ROS and display a greater dependency on UPR-activated mTOR signaling suggest a synthetic lethal vulnerability in *KRAS*-mutant cancer (Figs. 1, 2, 3). To test this hypothesis, we treated *NOP56* KD H358 and control cells with rapamycin, which, as expected, decreased mTOR effectors (e.g., p-S6, p-eIF4E) (Fig. 4A) that were the otherwise adaptively upregulated upon NOP56 depletion (Fig. 3G, H). Strikingly, rapamycin induced PARP cleavage (Cl PARP) in *NOP56* KD H358 but not in control cells (Fig. 4A), indicating that concomitant targeting of NOP56 and mTOR caused synthetic lethality. Similar results were observed in *NOP56* KD H358 cells that were treated with the AKT inhibitor AZD5363 (Fig. S4A, B). Knockdown of Raptor and Rictor, key components of the mTORC1 and mTORC2, respectively, significantly suppressed the viability of *NOP56* KD H358 cells despite to differential extent (Fig. S4C, D). Moreover, whereas individual S6 and eIF4E only partly contributed to the viability of *NOP56* KD H358 cells, concomitant inhibition of S6 (siRNA) and eIF4E (siRNA and Briciclib, an eIF4E inhibitor) led to significantly enhanced anti-proliferative effect (Fig. 4B-G; Fig. S4E) and largely recapitulated the impact seen by mTOR inhibition with rapamycin (Fig. 4A, Fig. S4F), gauged by the extent to which apoptotic markers (CI-PARP) were induced in *NOP56* KD H358 cells (Fig. 4F). These results interrogate an important role for mTOR to relay the IRE1α-mediated UPR signaling in NOP56-depleted *KRAS*-mutant lung cancer.

The UPR is a double-edged sword, as its outcome flips from adaption to apoptosis when malfunctional UPR, a condition at which stress stimuli are overwhelming or the UPR signal cannot be properly relayed [38, 39]. We thus assumed that the observed synthetic lethality of NOP56 and mTOR inhibition might be enabled due to malfunctional UPR. Indeed, co-targeting NOP56 and mTOR resulted in synergistic effects that not only increased the expression of IRE1α and p-IRE1α, indicative of hyperactive UPR signals, but also upregulated p-JNK, FOXO3A and BIM, a BH3 only protein and key mediator of apoptotic balance (Fig. 4H). Consistent with their pro-apoptotic roles, this increase of the JNK-FOXO3a-BIM axis was accompanied by PARP cleavage (Cl-PARP) and significantly greater apoptotic cell death in *NOP56* KD H358 and H460 cells compared to control cells (Fig. 4H, I). Importantly, IRE1α KD (siRNA) precluded the cytotoxicity of combined NOP56 and mTOR inhibition, evidenced by decreased levels of p-JNK, FOXO3a, BIM, Cl-PARP and of apoptotic populations in *NOP56* KD H358 and H460 treated with rapamycin (Fig. 4H, I). Similarly, inhibiting JNK activity by the inhibitor SP600125 dampened the efficacy of rapamycin in *NOP56* KD H358 and H460 cells (Fig. 4J). Thus, IRE1α-mediated UPR activates mTOR, which provides a survival signal for *NOP56* KD *KRAS*-mutant cancer cells; conversely, mTOR inhibition leads to overwhelmed UPR and promotes apoptotic cell death by activating the JNK-FOXO3A-BIM axis (Fig. 4K).

### NOP56 and mTOR converge on a metabolic liability in *KRAS*-mutant lung cancer

**Fig. 5:**
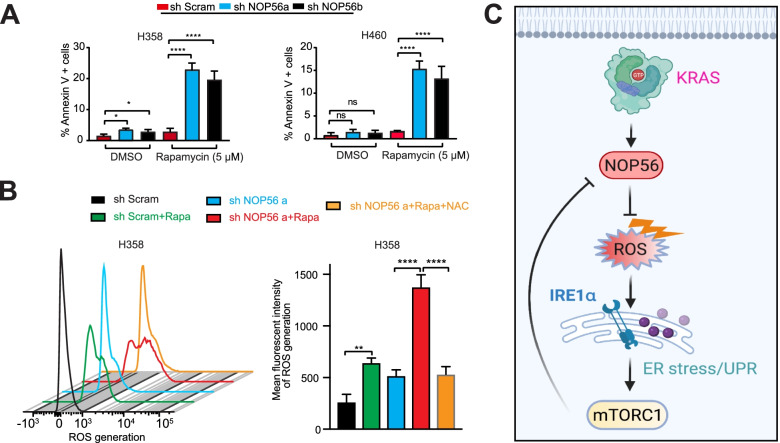
NOP56 and mTOR converge on a metabolic liability in *KRAS*-mutant tumor growth. **A**, Apoptosis assay of H358 and H460 cells expressing control or *NOP56*-specific shRNAs after treatment with rapamycin (5 μM) for 72 h. Data are presented as mean of three independent experiments (*n* = 3). *p < 0.05, ****P < 0.0001 and ns *P*>0.05 by two-way ANOVA with Tukey’s multiple comparisons test. **B**, H358 and H460 cells expressing control or *NOP56*-specific shRNAs were treated with rapamycin (5 μM) for 24 h. Cells were then washed, incubated with H2DCFDA for 30 min, and analyzed by flow cytometry. Quantification of relative ROS levels was shown in the right. **C**, A working model for the function of NOP56 in *KRAS*-mutant cancers

Next, we asked if the synthetic lethality of co-targeting NOP56 and mTOR is a result of unresolvable metabolic stress. Indeed, rapamycin sensitivity of *NOP56* KD H358 and H460 cells was highly correlated with ROS levels (Fig. 5A, B), and ROS scavenge by NAC significantly compromised the cytotoxicity of rapamycin (Fig. 5B), highlighting a causative link between ROS and rapamycin-induced apoptosis in *NOP56* KD cells.

These results uncover a novel homeostatic mechanism of metabolic stress mediated by NOP56 and validate an unexpected synthetic lethality by targeting NOP56 and mTOR that aggregate a metabolic liability in *KRAS*-mutant lung cancer (Fig. 5C).

### NOP56 downregulation plus rapamycin potently suppresses in vivo tumor growth of *KRAS*-mutant lung cancer

**Fig. 6:**
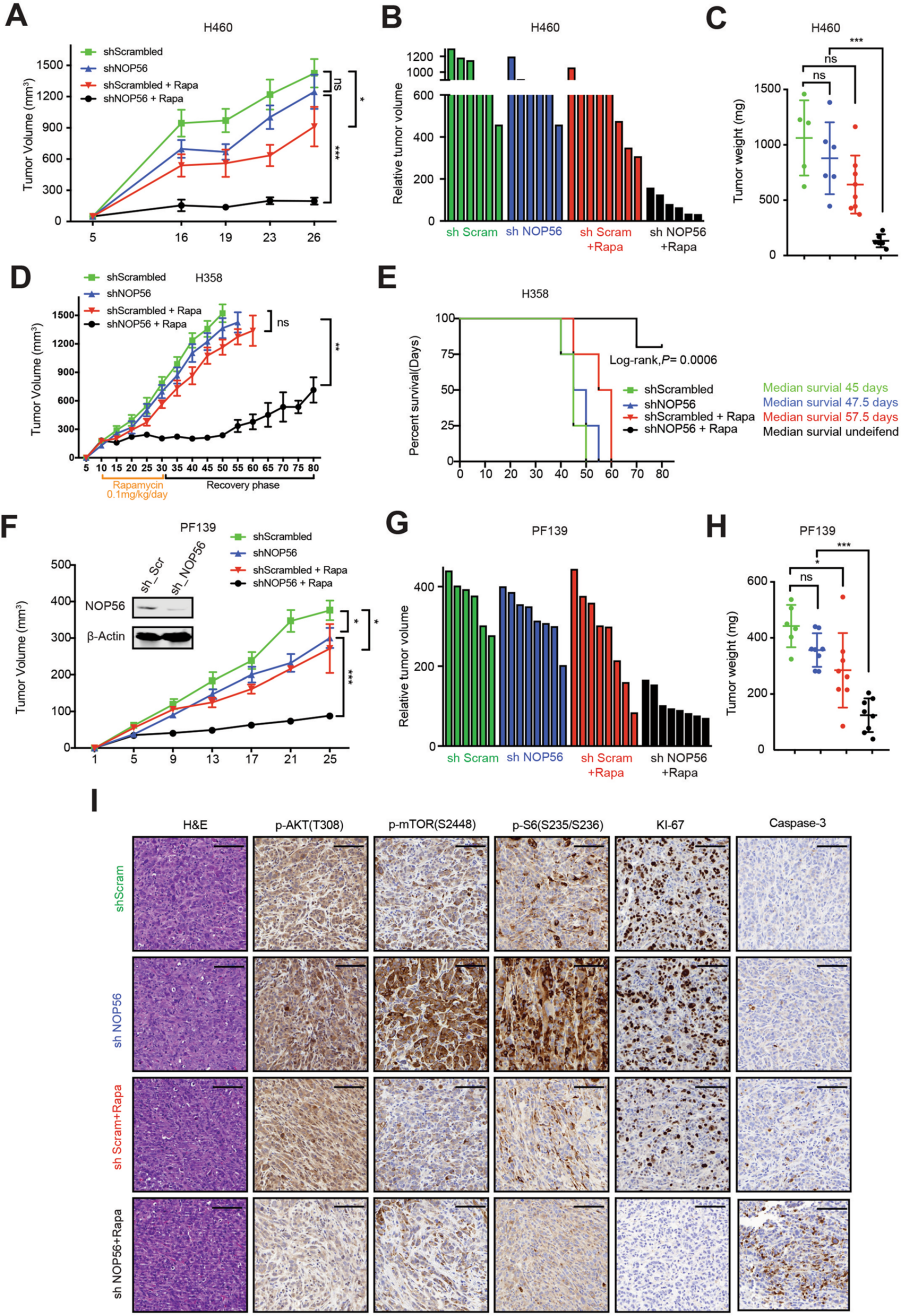
NOP56 knockdown plus rapamycin inhibits *KRAS*-mutant tumor growth. **A**, Growth curve of xenograft tumors derived from H460 cells expressing either a control or an shRNA against NOP56 (shNOP56a). Rapamycin (0.1 mg/kg) was administrated i.p. for 3 weeks (5 days/week). Data are shown as mean ± SD). ***P < 0.001, *P < 0.05 and ns (*P*>0.05) by two-way ANOVA with Tukey’s multiple comparisons test. **B**, Relative tumor volume of H460 xenograft tumors after the treatment for 3 weeks. **C**, Weights of H460 xenograft tumors after the treatment for 3 weeks. ***P < 0.001 by one-way ANOVA with Tukey’s multiple comparisons test. **D**, Growth curve of xenograft tumors derived from H358 cells expressing either a control or an shRNA against NOP56 (shNOP56a). **P < 0.01 by two-way ANOVA with Tukey’s multiple comparisons test. **E**, Kaplan-Meier survival curve of mice with H358 xenografts from the experiment in **D**. **F**, Growth curve of PDX tumors derived from primary *KRAS*-mutant PF139 lung cancer cells expressing either control or *NOP56*-specific shRNAs. Data are shown as mean ± SD). ***P < 0.001 and *P < 0.05 by two-way ANOVA with Tukey’s multiple comparisons test. Immunoblots of PF139 cells expressing *NOP56*-specific or scrambled shRNAs was also shown. **G**, Relative tumor volume of PF139 xenografts after 3 weeks of treatment. **H**, Weights of PF139 xenograft tumors after treated for 3 weeks. ***P < 0.001, *P < 0.05 and ns *P*>0.05 by two-way ANOVA with Tukey’s multiple comparisons test. **I**, H&E and IHC of p-AKT(T308), p-mTOR(S2448), p-S6(S235/236), Ki67 and Caspase-3) in PF139 xenograft tumors after the treatment. Scale bars 100 μm

Finally, we investigated in vivo efficacy of co-targeting NOP56 and mTOR. In a xenograft model from *KRAS*-mutant H460 cells, NOP56 knockdown (shNOP56) only mildly inhibited tumor growth compared to control shRNA (shScrambled), as did rapamycin (Fig. 6A). However, the outcome of concomitant targeting of NOP56 and mTOR (shNOP56 plus rapamycin) was superior to that achieved by shNOP56 or rapamycin alone, leading to far more effective and potent suppression of xenograft tumor growth (Fig. 6A, B). Residual tumors (after 3-week treatment) from the combination group (shNOP56 plus rapamycin) were typically tiny and significantly differed from those of the other treatment groups (Fig. 6C). Immunohistochemical (IHC) analysis revealed that the anti-tumor efficacy of combined treatment with shNOP56 and rapamycin was paralleled by marked decrease of mTOR activity (p-AKT, p-mTPOR, p-S6) and increase in apoptosis (Caspase-3) in the residual tumors (Fig. S5A).

Similar results were obtained from H358 xenografts (Fig. 6D, E) and a patient-derived xenograft (PDX) model established from the primary *KRAS*-mutant PF139 cells (Fig. 6F-I). In both models, *NOP56* KD sensitized H358 and PF139 xenograft tumors to rapamycin, leading to potent suppression of tumor growth (Fig. 6D, F-H) and significant improvement of mouse survival (Fig. 6E). IHC of the PF139 residual tumors revealed that NOP56 depletion plus rapamycin strikingly suppressed tumor cell proliferation (Ki-67) and dampened mTOR activity (p-AKT, p-mTOR and p-S6), but increased Caspase-3 cleavage (Fig. 6I), which is consistent with the in vitro results (Figs. 4, 5) and our observations on H460 xenografts (Fig. S5A). Notably, rapamycin showed little beneficial effects in *NOP56* KD *KRAS* wild-type H1703 xenografts (Fig. S5B, C), which mirrors the in vitro data and reinforces the selective activity of co-targeting NOP56/mTOR in *KRAS*-mutant lung cancer.

Together, these results support a model that NOP56 downregulation induces a metabolic vulnerability to mTOR inhibition, which presents a new and rational strategy for treating *KRAS-*mutant lung cancer.

## Discussion

In the present study, we have uncovered a new and unanticipated mechanism by which NOP56 and mTOR signaling cooperate in metabolic stress response in *KRAS*-mutant lung cancer. We show that NOP56 suppresses ROS, and its depletion induces synthetic lethal susceptibility to inhibition of mTOR that is otherwise essential for counterbalance of the resurge of cytotoxic ROS evoked by NOP56 downregulation. We also discover that mTOR activation is driven by IRE1α-mediated UPR via p38 MAPK. These findings support a model that NOP56 plays a role in the surveillance of ROS homeostasis and suggest that concomitant blockage of NOP56 and mTOR signaling has the potential to selectively target *KRAS*-mutant lung cancer. As multiple mTOR inhibitors (e.g., rapamycin and everolimus) are clinically approved drugs, our observations have immediate translational significance.

Despite decades-long steady efforts, therapeutic targeting of *KRAS*-mutant cancers has remained an overarching challenge in clinical oncology [3]. A promising strategy to target *KRAS*-driven tumors is to exploit cancer cell vulnerabilities contextually co-opted by mutant KRAS, in light of the concept that mutant KRAS alter physiological biochemical networks and induces cellular stresses, rendering *KRAS*-mutant cancer particularly susceptible to inhibition of stress-remedy mechanisms [12–14]. Empowered by CRISPR- and shRNA-based functional genomics, a plethora of novel factors required for *KRAS*-mutant cancer cells have been identified [12–16, 26], although the long-sought-after universal synthetic lethal targets for *KRAS-*driven pan-cancers are still at large. By implementing integrated analysis of functional genomic datasets (*n* = 5) derived from shRNA- and CRISPR-based screens [12, 16, 26], we revealed that NOP56 confers a metabolic requirement for *KRAS*-mutant cancer by regulating ROS homeostasis. A functional link between NOP56 and mutant KRAS is supported by a multitude of lines of evidence, i.e., the elevated expression of NOP56 in *KRAS*-mutant tumors, the prognostic significance of NOP56 expression in patients with *KRAS*-mutant but not wild-type cancers, and selective damage on *KRAS*-mutant cancer cells incurred by NOP56 downregulation.

Moreover, our results uncover a reciprocal interaction of NOP56 and mTOR signaling and suggest that combined inhibition of NOP56/mTOR is a rational strategy to combat *KRAS*-mutant cancer. Supporting our findings, mRNA levels of *NOP56* significantly correlate with that of mTOR pathway genes in *KRAS*-mutant cancer cells and lung adenocarcinomas, and *NOP56* expression is a predictive marker of sensitivity to mTOR inhibitors in *KRAS*-mutant but not *KRAS*-wild-type cancer cells. Importantly, *NOP56* knockdown sensitizes *KRAS*-mutant cancer cells to mTOR inhibitors in vitro and in vivo, which is not true for *KRAS*-wild-type tumor cells. Despite potential toxicological challenges as ribosome biogenesis is also an important physiological process, modulation of NOP56 activity may afford a therapeutic window for targeted inhibition of mTOR in *KRAS*-mutant cancers.

An increasingly growing body of evidence suggests that oncogenic KRAS signaling rewires metabolic pathways to meet the energetic and biosynthetic demands of cancer cells [27–30, 40]. In particular, increased ROS production, which has been shown to be functionally required for KRAS-mediated tumorigenicity [27, 28], is a key metabolic manifestation associated with *KRAS*-mutant cancer cells [27–30]. Since excess ROS is harmful, cancer cells must leverage ROS levels to favor tumor progression but prevent cell death [14, 27–30, 41]. Here, we reported, for the first time, a role for NOP56 in metabolic ROS response in *KRAS*-mutant lung cancer. NOP56 is a key component of box C/D snoRNPs that regulates ribosome assembly. This process has been shown to be deregulated in tumors with increased requirement for protein synthesis, providing cancer vulnerabilities for therapeutic avenues [21–24, 42–45]. Our results are also in line with previous studies reporting cancer subtype-specific alterations in ribosome assembly and biogenesis processes [44, 45]. In addition, recent evidence suggests that snoRNPs may also be involved in other processes independent of their functions in ribosome biogenesis [46]. Future studies will be necessary to clarify whether the newly identified metabolic role of NOP56 in *KRAS*-mutant cancer is related to its canonical role.

Our finding that IRE1α-mediated UPR connects the NOP56 function in ROS scavenge with mTOR signaling, a master regulator of cellular metabolism [47], provides mechanistic insights about the synthetic lethality of co-targeting NOP56 and mTOR, which is supported the observation that challenges to ribosome biogenesis result in acute loss of proteostasis [48]. IRE1α-mediated UPR, whilst initially protective, turns to be pro-apoptotic if ROS-induced metabolic stress is prolonged and persists [38, 39]. We further reveal that the overwhelming metabolic stress incurred by NOP56 depletion and mTOR inhibition activates the JNK-BIM axis, a stress-responsive pathway that promotes cell-cycle arrest and apoptosis [37]. The observation that UPR is a key component of the homeostatic mechanism in response to ROS resurge evoked by NOP56 knockdown is consistent with the notion that UPR plays an import role in homeostasis regulation including ROS and that challenges to ribosome biogenesis result in acute loss of proteostasis [49]. As metabolic ROS is causally linked to mutant KRAS-induced tumorigenicity and requires homeostatic mechanisms to maintain ROS levels within a threshold favorable for tumor development [14, 27–30, 41], the identification of NOP56 and mTOR converging on a role as ROS scavengers reveals an unanticipated metabolic vulnerability in *KRAS*-mutant cancers.

## Conclusion

In summary, we have uncovered an unexpected role for NOP56 in the surveillance of metabolic ROS in *KRAS*-mutant lung cancer. We have also revealed a novel synthetic lethality between NOP56 depletion and mTOR inhibitors that occurs by impeding the homeostatic mechanism of ROS in *KRAS*-mutant cancer cells. Moreover, we have demonstrated that mTOR activation upon NOP56 depletion is driven by IRE1α-mediated UPR. These results shed light on the mechanisms underlying KRAS-induced metabolic rewiring, reveals an unanticipated metabolic vulnerability in *KRAS*-mutant lung cancer, and suggest a new rationale for the treatment of the disease*.* Because *KRAS* alterations are implicated in a broad spectrum of human malignancies, our findings may also be applicable to other lineages of cancer with high frequencies of *KRAS* alterations.

## Supplementary Information

**Additional file 1: Figure S1.** NOP56 knockdown inhibits proliferation of *KRAS*-mutant cancer cells. **A**, Immunoblots of *KRAS*-mutant and *KRAS*-wild type cancer cells that were transfected with *NOP56*-specific siRNAs (si-*NOP56*) or scramble control siRNAs (si-Control). **B**, KRAS mutant and KRAS wild type cancer cells were transfected with control siRNAs or *NOP56*-specific siRNAs. Cell viability was determined 72 h post transfection. Data are presented as mean ± SD (*n* = 3). *p < 0.05, **p < 0.01, ***P < 0.001, ****P < 0.0001 and ns *P*>0.05 by two-way ANOVA with Tukey’s multiple comparisons test. **C**, *NOP56* is not a biomarker of survival in patients with *KRAS*-wild-type lung adenocarcinoma (LC), pancreatic cancer (PC) and colon cancer (CC). Kaplan–Meier survival analyses of patient cohorts in TCGA were stratified by the optimal cut-off value of the mRNA level of *NOP56*. **D**, Gene set enrichment analysis (GSEA) of a TCGA cohort of patients with *KRAS*-mutant lung (*n* = 141), pancreatic (*n* = 133) and colon cancer (*n* = 170). **Figure S2.** Stable expression of *NOP56-*specific shRNAs activates IRE1α-mediated UPR. **A**, Immunoblots of H358 and H460 cells expressing scrambled control or *NOP56* shRNAs. **B**, Immunofluorescence of H358 and H460 cells that express scrambled control or *NOP56* shRNAs. The NOP56 signal is indicated by arrowheads. **C**, The cell viability curve of H358 and H460 cells expressing scramble control shRNA or the *NOP56*-targeted shRNAs was measured at the indicated time points. **D**, Clongenic assay of H358 and H460 cells expressing scramble control or *NOP56*-targeted shRNAs. Quantification of clongenic assay were shown underneath. Data are presented as mean ± SD (*n* = 3). **E**, Growth inhibition of H358 and H460 cells expressing control shRNA or *NOP56*-targeted shRNA (3000 cells/well) treated for 72 h with the indicated doses of an IRE1α inhibitor (4μ8C). Data are presented as mean ± SD (*n* = 3). **F**, Apoptosis assay of H460 cells expressing scrambled control or *NOP56*-targeted shRNAs after transfection with *IRE1α-*specific or control siRNAs for 72 h. Data are presented as mean ± SD (*n* = 3). ***P < 0.001 and ns *P*>0.05 by two-way ANOVA with Tukey’s multiple comparisons test. **Figure S3.** NOP56 KD renders *KRAS*-mutant lung cancer cells susceptible to mTOR inhibition. **A**, Bar graphs illustrating the change of sensitivity to different inhibitors in H460 cells after *NOP56* knockdown. Data are presented as IC_50_ values of the indicated inhibitors in H460 cells expressing scramble control shRNAs compared to IC_50_ in H460 cells expressing *NOP56*-targeted shRNAs. Data are shown as mean (*n* = 2). **B**, Viability assay of H460 and H358 cells expressing control shRNA or NOP56-targeted shRNA (3000 cells/well) after treated for 72 h with the indicated doses of PI3K inhibitor (LY294002) and AKT inhibitor (AZD5363). Data are presented as mean ± SD (*n* = 3). **C**, Viability assay of H460 and H358 cells expressing control shRNA or NOP56-targeted shRNA (3000 cells/well) after treated for 72 h with the indicated doses of BiP inhibitor (HA15) and ER stress inducer (bortezomib). Data are presented as mean ± SD (*n* = 3). **D**, Immunoblots of *KRAS*-mutant (H358, H460) and wild-type (H1703, H520) cells expressing *NOP56*-specific sgRNAs. **E**, Viability assay of the cells expressing control or *NOP56*-specific sgRNAs after treated with rapamycin for 72 h. Data are shown as mean ± SD (*n* = 3). **F**, *NOP56* is negatively correlated with PI3K/AKT/mTOR pathway genes (*PI3KCA*, *PDPK1*, *PIK3R1*) in *KRAS*-mutant lung cancer patients. Pearson and Spearman coefficient and significance (*p*-value) are analyzed using R software (Cor.test function). **G**, Immunoblots of H460 and H1703 cells expressing control or *NOP56-*targeted shRNAs. **H**, **I**, Viability assay of *KRAS*-mutant (**H**) and wildtype (**I**) cancer cells expressing control or *NOP56*-specific siRNAs after treated with rapamycin. The assay was performed 72 h after drug treatment (96 h after siRNA transfection). **Figure S4.** NOP56 KD activates and induces dependence on the mTOR pathway in *KRAS*-mutant cancer cells. **A**, Immunoblots of H358 cells expressing control or *NOP56*-targeted shRNAs after treated with the AKT inhibitor (AZD5363) for 24 h. **B**, Clongenic assay of H358 cells expressing control or *NOP56*-specific shRNAs after treated with indicated doses of AZD5363. Representative images are shown. **C**, **D**, Immunoblots (**C**) and viability assay (**D**) of H358 cells expressing control or *NOP56-*specific shRNAs after transfected with *raptor*- or *rictor*-specific or control siRNAs for 72 h. Data are presented as mean ± SD (*n* = 3). **E**, Clongenic assay of H358 and H460 cells expressing control shRNA or *NOP56*-specific shRNAs after treatment with indicated doses of eIF4E inhibitor (Briciclib). Representative images are shown. **F**, Immunoblots of H460 cells expressing control or *NOP56*-target shRNAs after treated with rapamycin (1 μM) for 24 h. **Figure S5.** In vivo activity and selectivity of co-targeting NOP56 and mTOR in *KRAS*-mutant lung cancer. **A**, H&E and IHC analysis of p-AKT(T308), p-mTOR(S2448),p-S6(S235/236), Ki67 and Caspase-3) in residual H460 xenograft tumors after the indicated treatment. Scale bars 100 μm. **B**, Tumor volume of H1703 xenografts in immunocompromised (NSG) mice. H1703 cells were transduced with either a control or an shRNA against NOP56 (shNOP56a). Tumors were measured every 5 days with a caliper. **C**, Kaplan-Meier survival curve of mice harboring H1703 xenografts from the experiment shown in B.

**Additional file 2: Table S1.** Cell lines used in this study. **Table S2.** Inhibitors used for synthetic lethal chemical screens. **Table S3.** Antibodies used in this study. **Table S4.***KRAS* synthetic lethal (SL) genes.

## Data Availability

All data generated or analysed during this study are included in this published article and its supplementary information files.

## Abbreviations

GSEA: Gene Set Enrichment Analysis
IRE1α: Inositol-requiring enzyme 1α
KRAS: Kirsten rat sarcoma viral oncogene homolog
mTOR: Mechanistic target of rapamycin
NOP56: Nucleolar protein 5A
PARP: Poly (ADP-ribose) polymerase
RNAi: RNA interference
ROS: Reactive oxygen species
TCGA: The Cancer Genome Atlas
UPR: Unfolded protein response
