# Supplementary material for: Metabolic synthetic lethality by targeting NOP56 and mTOR in KRAS-mutant lung cancer
Source: J Exp Clin Cancer Res. 2022 Jan 17;41:25. doi: 10.1186/s13046-022-02240-5 (PMC8762933; doi:10.1186/s13046-022-02240-5)
Supplement: Supplementary file 1 — Additional file 1: Figure S1. NOP56 knockdown inhibits proliferation of KRAS-mutant cancer cells. A, Immunoblots of KRAS-mutant and KRAS-wild type cancer cells that were transfected with NOP56-specific siRNAs (si-NOP56) or scramble control siRNAs (si-Control). B, KRAS mutant and KRAS wild type cancer cells were transfected with control siRNAs or NOP56-specific siRNAs. Cell viability was determined 72 h post transfection. Data are presented as mean ± SD (n = 3). *p < 0.05, **p < 0.01, ***P < 0.001, ****P < 0.0001 and ns P>0.05 by two-way ANOVA with Tukey’s multiple comparisons test. C, NOP56 is not a biomarker of survival in patients with KRAS-wild-type lung adenocarcinoma (LC), pancreatic cancer (PC) and colon cancer (CC). Kaplan–Meier survival analyses of patient cohorts in TCGA were stratified by the optimal cut-off value of the mRNA level of NOP56. D, Gene set enrichment analysis (GSEA) of a TCGA cohort of patients with KRAS-mutant lung (n = 141), pancreatic (n = 133) and colon cancer (n = 170). Figure S2. Stable expression of NOP56-specific shRNAs activates IRE1α-mediated UPR. A, Immunoblots of H358 and H460 cells expressing scrambled control or NOP56 shRNAs. B, Immunofluorescence of H358 and H460 cells that express scrambled control or NOP56 shRNAs. The NOP56 signal is indicated by arrowheads. C, The cell viability curve of H358 and H460 cells expressing scramble control shRNA or the NOP56-targeted shRNAs was measured at the indicated time points. D, Clongenic assay of H358 and H460 cells expressing scramble control or NOP56-targeted shRNAs. Quantification of clongenic assay were shown underneath. Data are presented as mean ± SD (n = 3). E, Growth inhibition of H358 and H460 cells expressing control shRNA or NOP56-targeted shRNA (3000 cells/well) treated for 72 h with the indicated doses of an IRE1α inhibitor (4μ8C). Data are presented as mean ± SD (n = 3). F, Apoptosis assay of H460 cells expressing scrambled control or NOP56-targeted shRNAs after transfection [file 13046_2022_2240_MOESM1_ESM.pdf]

Supplementary Information

# Metabolic synthetic lethality by targeting NOP56 and mTOR signaling in *KRAS*-mutant lung cancer

Zhang Yang[1¶], Shun-Qing Liang[1¶#], Liang Zhao[1], Haitang Yang[1§], Thomas M. Marti[1], Balazs Hegedüs[2], Yanyun Gao[1], Bin Zheng[3], Chun Chen[3], Wenxiang Wang[4], Patrick Dorn[1], Gregor J. Kocher[1], Ralph A. Schmid[1*], Ren-Wang Peng[1*]

## This PDF files include:

1. Supplementary Figures S1-S5

2. Supplementary Tables

   Table S1. Cell lines used in this study

   Table S2. Inhibitors used for synthetic lethal chemical screens

   Table S3. Antibodies used in this study

   Table S4. *KRAS* synthetic lethal (SL) genes

Figure S1

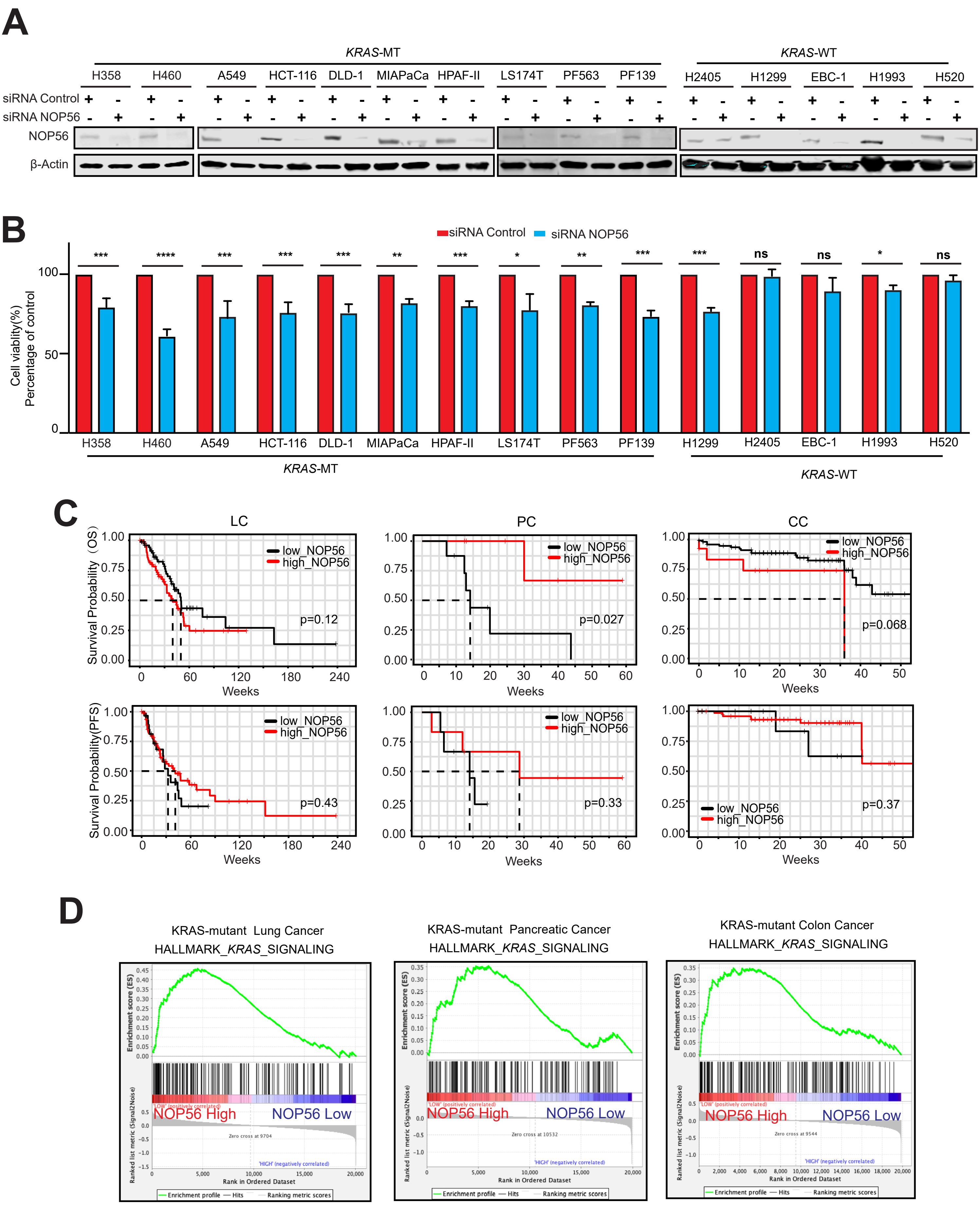

A
KRAS-MT
KRAS-WT
H358
H460
A549
HCT-116
DLD-1
MIAPaCa
HPAF-II
LS174T
PF563
PF139
H2405
H1299
EBC-1
H1993
H520
siRNA Control
siRNA NOP56
NOP56
β-Actin
B
siRNA Control
siRNA NOP56
Cell viablity(%)
Percentage of control
*** **** *** *** *** ** *** * ** *** *** ns ns * ns
KRAS-MT
KRAS-WT
C
LC
PC
CC
Survival Probability (OS)
Survival Probability(PFS)
low_NOP56
high_NOP56
p=0.12
p=0.027
p=0.068
p=0.43
p=0.33
p=0.37
Weeks
D
KRAS-mutant Lung Cancer
HALLMARK_KRAS_SIGNALING
KRAS-mutant Pancreatic Cancer
HALLMARK_KRAS_SIGNALING
KRAS-mutant Colon Cancer
HALLMARK_KRAS_SIGNALING
Enrichment score (ES)
Ranked list metric (Signal2Noise)
NOP56 High
NOP56 Low
Zero cross at 9704
Zero cross at 10532
Zero cross at 9544
Rank in Ordered Dataset
Enrichment profile
Hits
Ranking metric scores

**Figure S1: NOP56 knockdown inhibits proliferation of *KRAS*-mutant cancer cells.**

**A**, Immunoblots of *KRAS*-mutant and *KRAS*-wild type cancer cells that were transfected with *NOP56*-specific siRNAs (si-*NOP56*) or scramble control siRNAs (si-Control).

**B,** KRAS mutant and KRAS wild type cancer cells were transfected with control siRNAs or *NOP56*-specific siRNAs. Cell viability was determined 72 hours post transfection. Data are presented as mean ± SD (n=3). *$p$<0.05, **$p$<0.01, ***$P$<0.001, ****$P$<0.0001 and ns $P > 0.05$ by two-way ANOVA with Tukey's multiple comparisons test.

C, *NOP56* is not a biomarker of survival in patients with *KRAS*-wild-type lung adenocarcinoma (LC), pancreatic cancer (PC) and colon cancer (CC). Kaplan–Meier survival analyses of patient cohorts in TCGA were stratified by the optimal cut-off value of the mRNA level of *NOP56*.

**D,** Gene set enrichment analysis (GSEA) of a TCGA cohort of patients with *KRAS*-mutant lung (n=141), pancreatic (n=133) and colon cancer (n=170).

# Figure S2

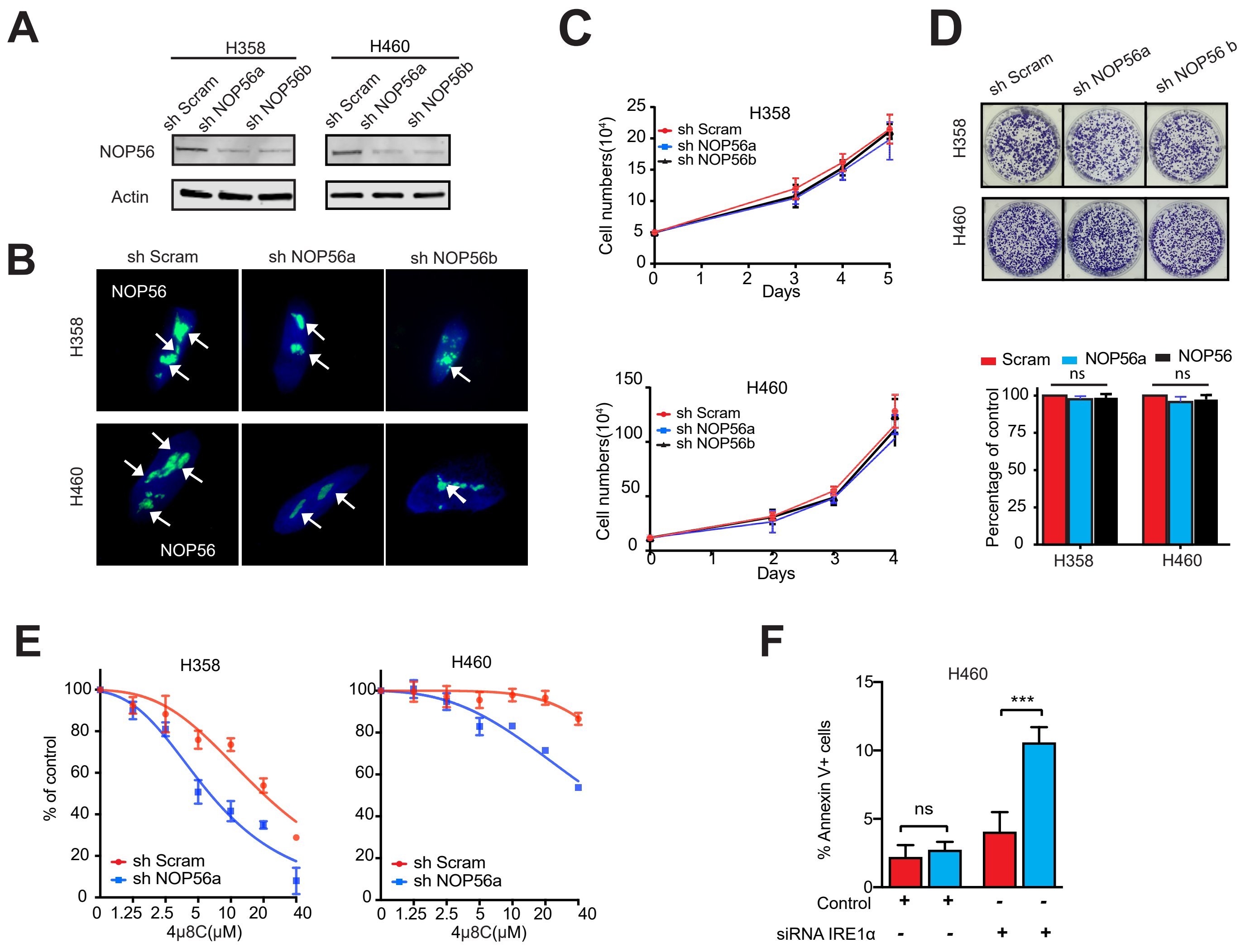
A
H358
H460
sh Scram
sh NOP56a
sh NOP56b
NOP56
Actin
B
sh Scram
sh NOP56a
sh NOP56b
NOP56
H358
H460
C
H358
Cell numbers($10^4$)
Days
sh Scram
sh NOP56a
sh NOP56b
H460
D
sh Scram
sh NOP56a
sh NOP56 b
Scram
NOP56a
NOP56
ns
Percentage of control
E
H358
H460
% of control
sh Scram
sh NOP56a
4μ8C(μM)
F
H460
% Annexin V+ cells
ns
***
Control
siRNA IRE1α

**Figure S2: Stable expression of *NOP56*-specific shRNAs activates IRE1α-mediated UPR**

**A**, Immunoblots of H358 and H460 cells expressing scrambled control or *NOP56* shRNAs.

**B**, Immunofluorescence of H358 and H460 cells that express scrambled control or *NOP56* shRNAs. The NOP56 signal is indicated by arrowheads.

**C,** The cell viability curve of H358 and H460 cells expressing scramble control shRNA or the *NOP56*-targeted shRNAs was measured at the indicated time points.

**D,** Clongenic assay of H358 and H460 cells expressing scramble control or *NOP56*-targeted shRNAs. Quantification of clongenic assay were shown underneath. Data are presented as mean ± SD (n=3).

**E,** Growth inhibition of H358 and H460 cells expressing control shRNA or *NOP56*-targeted shRNA (3000 cells/well) treated for 72h with the indicated doses of an IRE1α inhibitor (4μ8C). Data are presented as mean ± SD (n=3).

**F**, Apoptosis assay of H460 cells expressing scrambled control or *NOP56*-targeted shRNAs after transfection with *IRE1α*-specific or control siRNAs for 72 h. Data are presented as mean ± SD (n=3). ***$P$<0.001 and ns $P > 0.05$ by two-way ANOVA with Tukey's multiple comparisons test.

# Figure S3

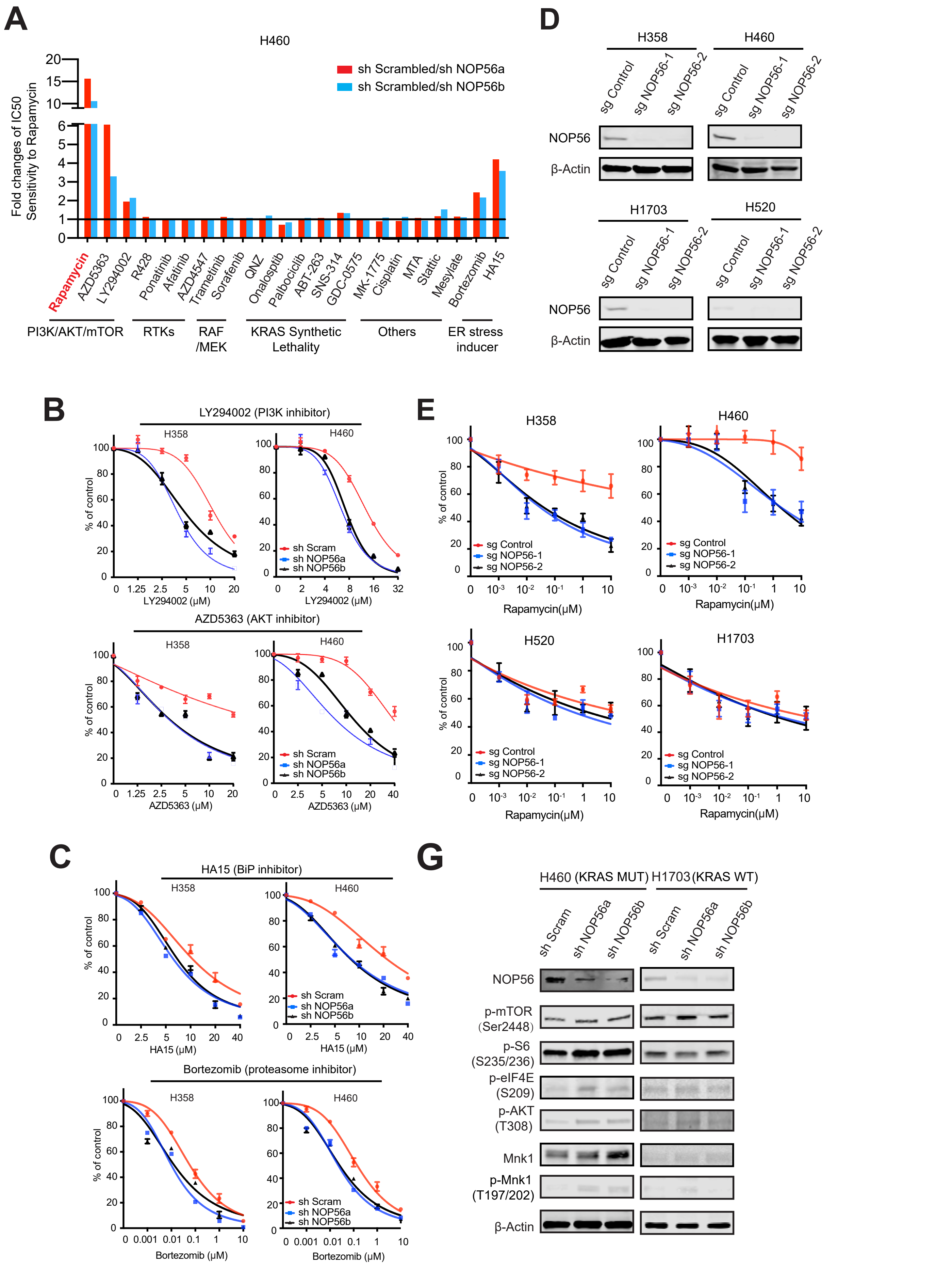
A
H460
Fold changes of IC50 Sensitivity to Rapamycin
sh Scrambled/sh NOP56a
sh Scrambled/sh NOP56b
Rapamycin
AZD5363
LY294002
R428
Ponatinib
Afatinib
AZD4547
Trametinib
Sorafenib
QNZ
Onalospib
Palbociclib
ABT-263
SNS-314
GDC-0575
MK-1775
Cisplatin
MTA
Stattic
Mesylate
Bortezomib
HA15
PI3K/AKT/mTOR
RTKs
RAF /MEK
KRAS Synthetic Lethality
Others
ER stress inducer
B
LY294002 (PI3K inhibitor)
H358
H460
% of control
LY294002 (μM)
sh Scram
sh NOP56a
sh NOP56b
AZD5363 (AKT inhibitor)
AZD5363 (μM)
C
HA15 (BiP inhibitor)
HA15 (μM)
Bortezomib (proteasome inhibitor)
Bortezomib (μM)
D
H358
H460
H1703
H520
sg Control
sg NOP56-1
sg NOP56-2
NOP56
β-Actin
E
H358
H460
H520
H1703
sg Control
sg NOP56-1
sg NOP56-2
Rapamycin(μM)
G
H460 (KRAS MUT)
H1703 (KRAS WT)
sh Scram
sh NOP56a
sh NOP56b
NOP56
p-mTOR (Ser2448)
p-S6 (S235/236)
p-eIF4E (S209)
p-AKT (T308)
Mnk1
p-Mnk1 (T197/202)
β-Actin

# Figure S3

F

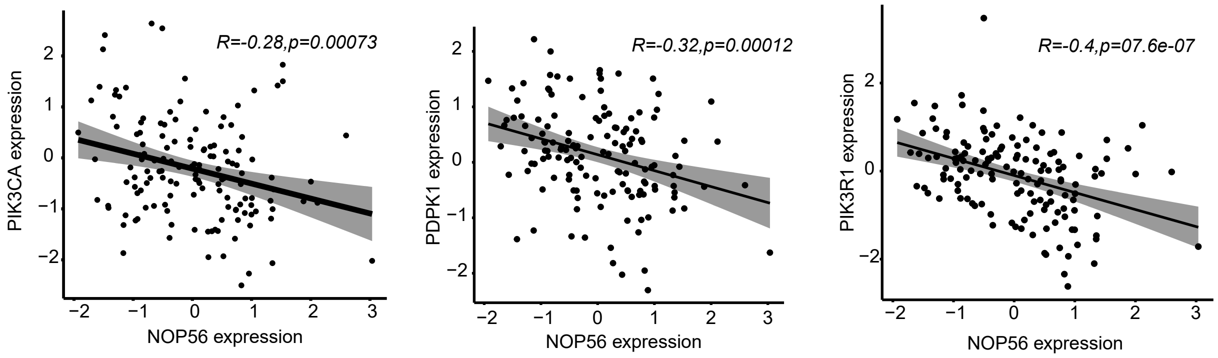


H

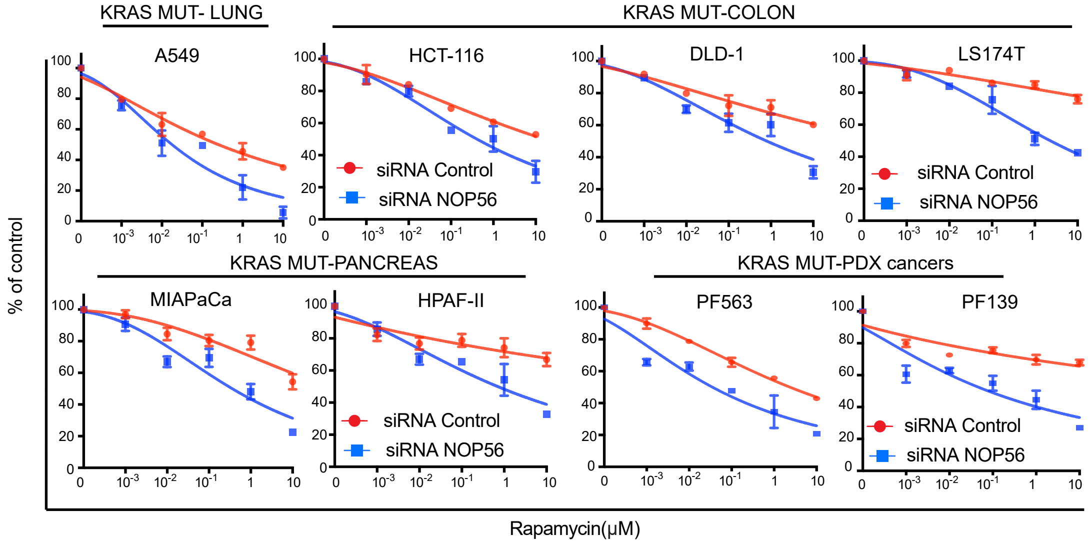


I

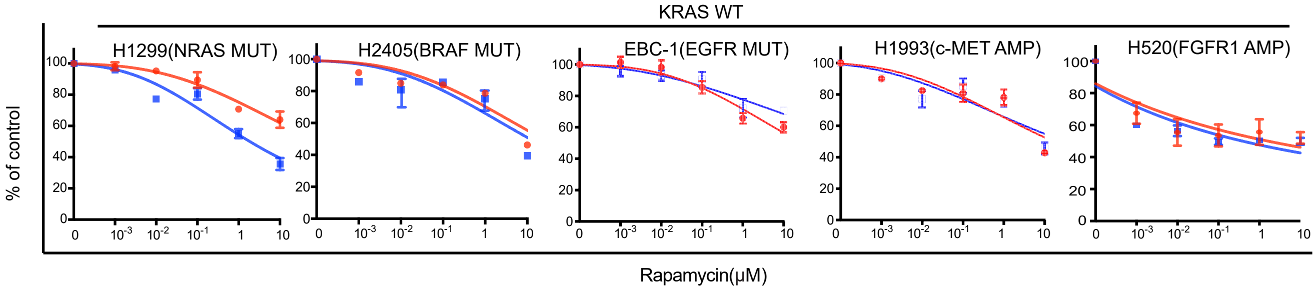

**Figure S3: NOP56 KD renders *KRAS*-mutant lung cancer cells susceptible to mTOR inhibition.**

**A,** Bar graphs illustrating the change of sensitivity to different inhibitors in H460 cells after *NOP56* knockdown. Data are presented as $IC_{50}$ values of the indicated inhibitors in H460 cells expressing scramble control shRNAs compared to $IC_{50}$ in H460 cells expressing *NOP56*-targeted shRNAs. Data are shown as mean (n=2).

**B,** Viability assay of H460 and H358 cells expressing control shRNA or NOP56-targeted shRNA (3000 cells/well) after treated for 72 h with the indicated doses of PI3K inhibitor (LY294002) and AKT inhibitor (AZD5363). Data are presented as mean ± SD (n=3).

**C,** Viability assay of H460 and H358 cells expressing control shRNA or NOP56-targeted shRNA (3000 cells/well) after treated for 72 h with the indicated doses of BiP inhibitor (HA15) and ER stress inducer (bortezomib). Data are presented as mean ± SD (n=3).

**D,** Immunoblots of *KRAS*-mutant (H358, H460) and wild-type (H1703, H520) cells expressing *NOP56*-specific sgRNAs.

**E,** Viability assay of the cells expressing control or *NOP56*-specific sgRNAs after treated with rapamycin for 72 h. Data are shown as mean ± SD (n=3).

**F,** *NOP56* is negatively correlated with PI3K/AKT/mTOR pathway genes (*PI3KCA*, *PDPK1*, *PIK3R1*) in *KRAS*-mutant lung cancer patients. Pearson and Spearman coefficient and significance (p-value) are analyzed using R software (Cor.test function).

**G,** Immunoblots of H460 and H1703 cells expressing control or *NOP56*-targeted shRNAs.

**H, I,** Viability assay of *KRAS*-mutant (**H**) and wildtype (**I**) cancer cells expressing control or *NOP56*-specific siRNAs after treated with rapamycin. The assay was performed 72 h after drug treatment (96h after siRNA transfection).

# Figure S4

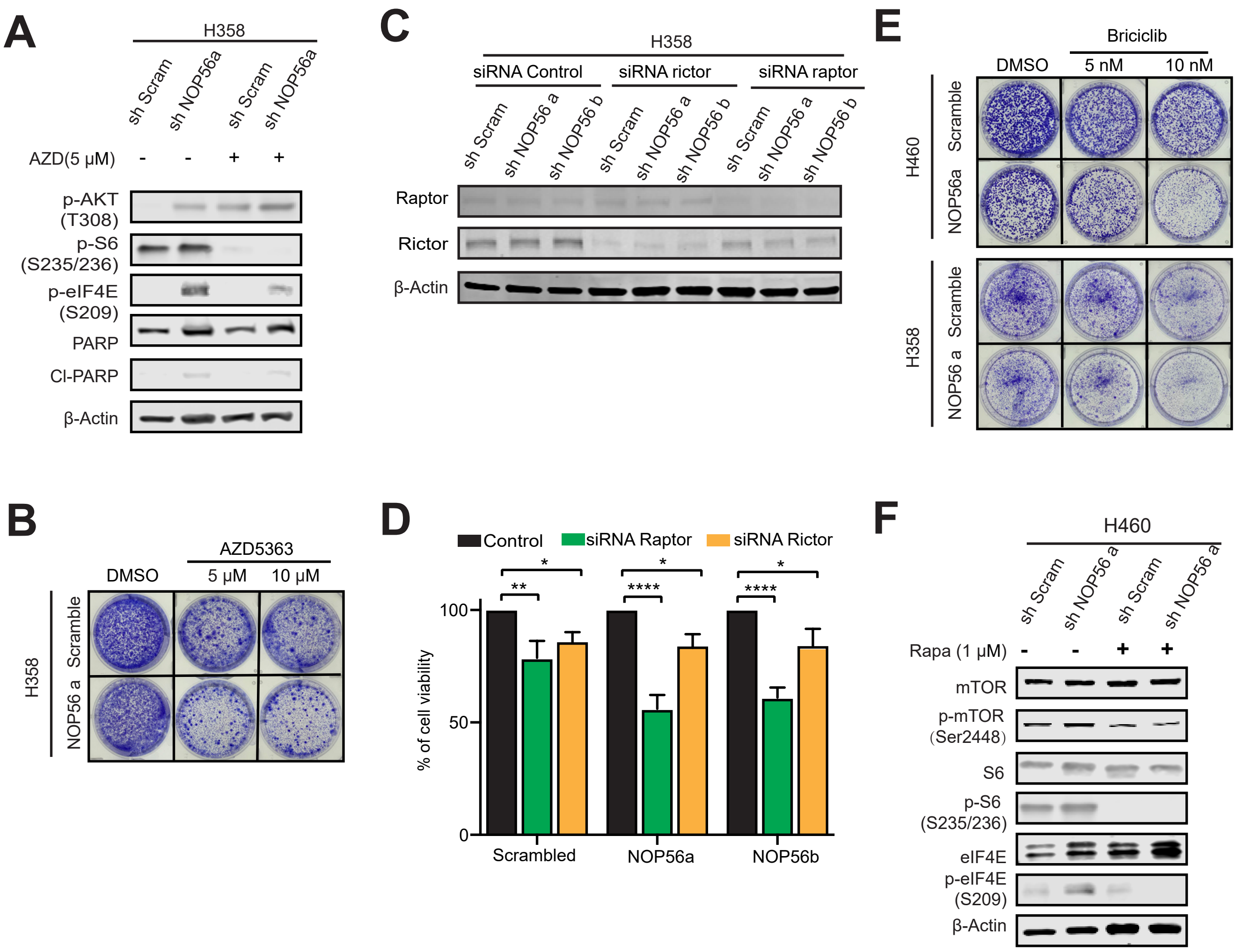

A
H358
sh Scram
sh NOP56a
sh Scram
sh NOP56a
AZD(5 μM) - - + +
p-AKT (T308)
p-S6 (S235/236)
p-eIF4E (S209)
PARP
Cl-PARP
β-Actin
B
AZD5363
DMSO
5 μM
10 μM
H358
Scramble
NOP56 a
C
H358
siRNA Control
siRNA rictor
siRNA raptor
sh Scram
sh NOP56 a
sh NOP56 b
Raptor
Rictor
β-Actin
D
Control
siRNA Raptor
siRNA Rictor
% of cell viability
100
50
0
**
*
****
Scrambled
NOP56a
NOP56b
E
Briciclib
DMSO
5 nM
10 nM
H460
H358
Scramble
NOP56a
NOP56 a
F
H460
sh Scram
sh NOP56 a
Rapa (1 μM) - - + +
mTOR
p-mTOR (Ser2448)
S6
p-S6 (S235/236)
eIF4E
p-eIF4E (S209)
β-Actin

**Figure S4: NOP56 KD activates and induces dependence on the mTOR pathway in *KRAS*-mutant cancer cells.**

**A,** Immunoblots of H358 cells expressing control or *NOP56*-targeted shRNAs after treated with the AKT inhibitor (AZD5363) for 24 h.

**B,** Clongenic assay of H358 cells expressing control or *NOP56*-specific shRNAs after treated with indicated doses of AZD5363. Representative images are shown.

**C, D,** Immunoblots (C) and viability assay (D) of H358 cells expressing control or *NOP56*-specific shRNAs after transfected with *raptor*- or *rictor*-specific or control siRNAs for 72 h. Data are presented as mean ± SD (n=3).

**E,** Clongenic assay of H358 and H460 cells expressing control shRNA or *NOP56*-specific shRNAs after treatment with indicated doses of eIF4E inhibitor (Briciclib). Representative images are shown.

**F**, Immunoblots of H460 cells expressing control or *NOP56*-target shRNAs after treated with rapamycin (1 μM) for 24 h.

# Figure S5

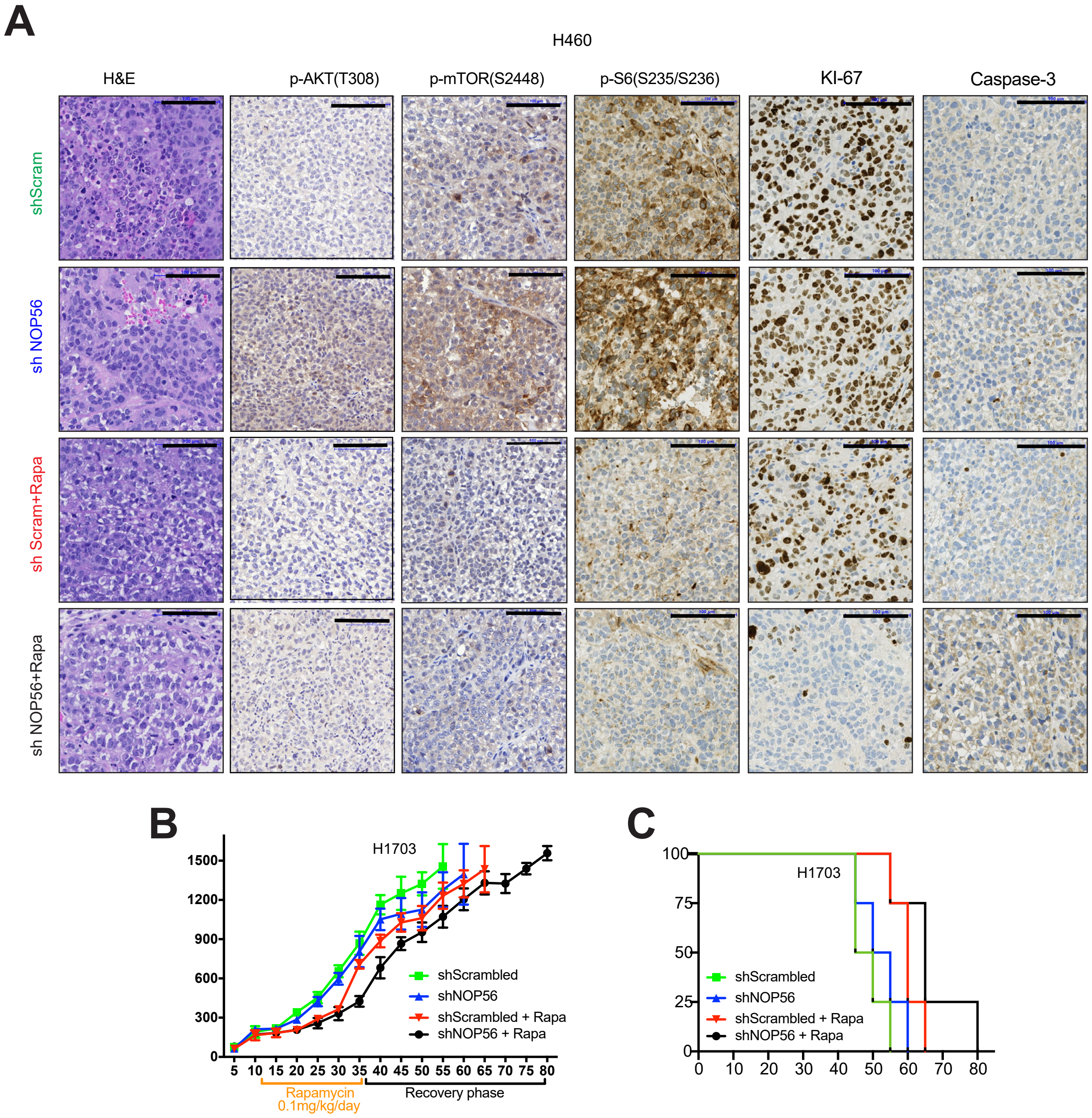
A
H460
H&E
p-AKT(T308)
p-mTOR(S2448)
p-S6(S235/S236)
KI-67
Caspase-3
shScram
sh NOP56
sh Scram+Rapa
sh NOP56+Rapa
B
H1703
1500
1200
900
600
300
0
5 10 15 20 25 30 35 40 45 50 55 60 65 70 75 80
Rapamycin
0.1mg/kg/day
Recovery phase
shScrambled
shNOP56
shScrambled + Rapa
shNOP56 + Rapa
C
H1703
100
75
50
25
0
0 10 20 30 40 50 60 70 80
shScrambled
shNOP56
shScrambled + Rapa
shNOP56 + Rapa

**Figure S5: *In vivo* activity and selectivity of co-targeting NOP56 and mTOR in *KRAS*-mutant lung cancer**

**A,** H&E and IHC analysis of p-AKT(T308), p-mTOR(S2448),p-S6(S235/236), Ki67 and Caspase-3) in residual H460 xenograft tumors after the indicated treatment. Scale bars 100 μm

**B,** Tumor volume of H1703 xenografts in immunocompromised (NSG) mice. H1703 cells were transduced with either a control or an shRNA against NOP56 (shNOP56a). Tumors were measured every 5 days with a caliper.

**C,** Kaplan-Meier survival curve of mice harboring H1703 xenografts from the experiment shown in B
